# Multiple Adaptive Strategies of Himalayan *Iodobacter* sp. PCH194 to High-Altitude Stresses

**DOI:** 10.3389/fmicb.2022.881873

**Published:** 2022-07-06

**Authors:** Vijay Kumar, Prakriti Kashyap, Subhash Kumar, Vikas Thakur, Sanjay Kumar, Dharam Singh

**Affiliations:** ^1^Biotechnology Division, CSIR-Institute of Himalayan Bioresource Technology, Palampur, India; ^2^Academy of Scientific and Innovative Research (AcSIR), CSIR–Human Resource Development Centre (CSIR-HRDC), Ghaziabad, India

**Keywords:** high-altitude Himalaya, bacterial adaptation, genomic traits, proteomic response, antifreeze proteins, polyhydroxybutyrate

## Abstract

Bacterial adaption to the multiple stressed environments of high-altitude niches in the Himalayas is intriguing and is of considerable interest to biotechnologists. Previously, we studied the culturable and unculturable metagenome microbial diversity from glacial and kettle lakes in the Western Himalayas. In this study, we explored the adaptive strategies of a unique Himalayan eurypsychrophile *Iodobacter* sp. PCH194, which can synthesize polyhydroxybutyrate (PHB) and violacein pigment. Whole-genome sequencing and analysis of *Iodobacter* sp. PCH194 (4.58 Mb chromosome and three plasmids) revealed genetic traits associated with adaptive strategies for cold/freeze, nutritional fluctuation, defense against UV, acidic pH, and the kettle lake's competitive environment. Differential proteome analysis suggested the adaptive role of chaperones, ribonucleases, secretion systems, and antifreeze proteins under cold stress. Antifreeze activity inhibiting the ice recrystallization at −9°C demonstrated the bacterium's survival at subzero temperature. The bacterium stores carbon in the form of PHB under stress conditions responding to nutritional fluctuations. However, violacein pigment protects the cells from UV radiation. Concisely, genomic, proteomic, and physiological studies revealed the multiple adaptive strategies of Himalayan *Iodobacter* to survive the high-altitude stresses.

## Introduction

The Himalayas host a range of environmental niches such as permafrost, glacial streams, lakes, sediments, meadows, and deserts that contain distinct microbial communities (Stres et al., 2013; Kumar et al., 2018, 2019, 2022; Thakur et al., 2018; Dhakar and Pandey, 2020). High-altitude Himalayan environments are characterized by multiple stress factors such as high UV irradiation, oxidative stress, frequent freeze-thaw cycles, desiccation, oxygen limitation, nutritional fluctuation, and perennially cold temperatures (Singh and Singh, 2004; Stres et al., 2013). Microbes thriving in permanently cold environments have evolved many adaptive strategies at the molecular and physiological levels (Mykytczuk et al., 2013; De Maayer et al., 2014; Goordial et al., 2016; Kumar et al., 2020a). Thus, the microbes thriving in high-altitude Himalayan niches provide an interesting model system to study microbial adaptation strategies in response to the fluctuating environment.

Global warming has led to the rapid meltdown and receding of Himalayan glaciers (Bolch et al., 2012; Maurer et al., 2019), resulting in new environmental niches that provide opportunities to study their microbial ecology (Hotaling et al., 2017). Glacial meltdown forms many enclosed depressions in a geographic area called kettle holes or kettle lakes (Corti et al., 2012). In fact, kettle lakes are exposed to frequent changes in physicochemical factors such as thermal, hydrodynamics, and nutrients (Reverey et al., 2018). Hence, such niches are a goldmine for studying biogeochemical cycles (Reverey et al., 2016, 2018), distinct microbial communities (Kumar et al., 2022), and commercially important bacteria (Kumar et al., 2021).

A recent glacial retreat has created a kettle lake at 4,200 m above sea level in the Sach Pass area of the Western Himalayas (Himachal Pradesh, India). The kettle lake is exposed to daily and seasonal fluctuations in thermal, hydrological, nutritional, and other physicochemical factors. The eurypsychrophilic bacterial strain *Iodobacter* sp. PCH194, which coproduces PHB and violacein pigment, was isolated from a sediment sample of this lake (Kumar et al., 2021). The genus *Iodobacter* is a minor member of microbial communities inhabiting rivers, streams, and low-temperature niches such as Arctic glacial lake sediment and the Antarctic Peninsula (Logan, 1989; Leblanc et al., 2009; Su et al., 2013; Atalah et al., 2020). *Iodobacter* and other violacein-producing bacteria are distinct in cold-water bodies and glacial environments. There are reports on violacein-producing bacteria from cold niches, studying the adaptive and ecological function of violacein pigment (Matz et al., 2004; Pantanella et al., 2007; Deines et al., 2009; Abboud and Arment, 2013; Mojib et al., 2013; Atalah et al., 2020). However, the unique features displayed by Himalayan *Iodobacter*, such as coproducing PHB and violacein and surviving in the freezing environment of high-altitude kettle lake, intrigue to explore its survival strategies.

This study investigated the adaptive strategies of Himalayan *Iodobacter* sp. PCH194 using genomic, proteomic, and physiological approaches. The bacterium possesses multiple genomic traits to cope with cold and freezing conditions, oxygen depletion, oxidative stress, starvation, acidic pH, and high UV irradiance in its high-altitude niche. Differential proteome and antifreeze activity analysis provides insight into a bacterial response to cold and freezing. In addition, physiological experiments demonstrated polyhydroxyalkanoate synthesis and violacein pigment-containing microbial mat formation as a key survival strategy. Our findings identify the multiple adaptive strategies of *Iodobacter* sp. PCH194 enable it to survive in the kettle lake environment of the high-altitude Himalaya.

## Materials and Methods

### Phenotypic and Biochemical Characterization of *Iodobacter* sp. PCH194

*Iodobacter* sp. PCH194, isolated previously from high-altitude kettle lake sediment (Kumar et al., 2021), was used for the study. The pure culture was submitted in patent deposit to the Microbial Type Culture Collection (MTCC number 25171) at CSIR-IMTECH, Chandigarh, India. The colony morphology of the bacterium was observed after 72 h of agar plate incubation at 20°C on the Antarctic bacterial medium (AMB) (0.5% peptone, 0.2% yeast extract, and 2.0% agar). The carbohydrate and sugar utilization tests and catalase, oxidase, and nitrite reduction activity assays were performed using HiMedia kits (HiMedia, Mumbai, India).

### Whole-Genome Sequencing and Genomic Insights of *Iodobacter* sp. PCH194

Genomic DNA isolation and library construction for *Iodobacter* sp. PCH194 were performed as described previously (Kumar et al., 2020a). The genomic library was evaluated using Qubit 2.0 (Invitrogen, Carlsbad, California, USA) and Bioanalyser (Agilent Technologies, Santa Clara, California, USA). Whole-genome sequencing (WGS) was carried out using the PacBio RSII system (PacBio, Menlo Park, California, USA), and genome assembly was performed using the RS Hierarchical Genome Assembly Process (HGAP) protocol version 3.0 (Chin et al., 2013). The complete genome sequence was submitted to the NCBI database with genome assembly accession number GCA_004194535.1, BioSample ID SAMN08324479, and BioProject ID PRJNA428922. Genome annotation was performed using Rapid Annotation and Subsystem Technology (RAST) (Aziz et al., 2008) and Prokaryotic Genome Annotation Pipeline (PGAP) version 3.1 (Tatusova et al., 2016). DNA-DNA hybridization (DDH) and average nucleotide identity (ANI) analyses were performed using online tools, *viz.*, Genome-to-Genome Distance Calculator (GGDC) (http://ggdc.dsmz.de/) (Meier-Kolthoff et al., 2013) and ANI calculator of Kostas lab (http://enve-omics.ce.gatech.edu/ani/) (Goris et al., 2007), respectively. Phylogenetic trees for the members of the genus *Iodobacter* were constructed based upon the available whole-genome sequences in the NCBI genome database. A phylogenetic tree was constructed using Type (Strain) Genome Server (TYGS) (https://tygs.dsmz.de/) by manually restricting the job to the input sequences (Meier-Kolthoff and Göker, 2019). Genes associated with adaptive traits such as carbon storage, respiration, chemotaxis, cold stress and oxidative stress response, acidic pH adaptation, defense against UV, and interspecies competition were mined by analyzing subsystem categories in RAST and NCBI PGAP files (Aziz et al., 2008; Tatusova et al., 2016).

### Growth of *Iodobacter* sp. PCH194 at low Temperatures

Growth of *Iodobacter* sp. PCH194 was monitored in nutrient broth medium (g/l; meat extract 1.0, peptone 5.0, yeast extract 2.0, NaCl 5.0) at a temperature range of −5 to 37°C by observing the OD_600_ and OD_460_. The specific growth rate (μ) of the bacterium was calculated in a static condition at temperatures of 0, 4, and 20°C and in a shaking condition at 20°C.

### Proteomic Analysis of *Iodobacter* sp. PCH194 Growing at Different Temperatures

The bacterium culture was inoculated in nutrient broth and incubated at 20°C to prepare a seed culture of 1.0 OD_460_. Seed culture (1.0%, v/v) was transferred to the same medium and incubated at temperatures of 20, 10, and 4°C. After 3 days of cold acclimation at 4°C (OD_460_ 0.567), the culture was transferred to 0°C and the growth was monitored. To further evaluate the adaptive changes, a differential proteome study was performed.

### Protein Extraction and In-Gel Digestion and MALDI-TOF-TOF Identification

*Iodobacter* sp. PCH194 was grown at 0, 4, and 20°C, and the culture was centrifuged at 9,600 *g* for 10 min to obtain the pellet. The bacterial pellet was resuspended in 10 mM Tris-HCl, pH 8.0, and total protein was extracted by sonicating the bacterial pellet. The protein was quantified using the Bradford assay with a standard curve of BSA at various concentrations. An equal amount of protein was loaded onto the 10% SDS PAGE to visualize differentially expressed proteins. The differentially expressed polypeptides from SDS-PAGE were excised, washed in deionized water, and destained in 100 mM NH_4_HCO_3_/50% acetonitrile for 15 min at room temperature (RT). Polypeptides were reduced with 10 mM dithiothreitol and alkylated with 55 mM iodoacetamide. The resulting alkylated polypeptides were dehydrated using acetonitrile followed by overnight digestion with a freshly prepared trypsin solution (25 mM NH_4_HCO_3_ with 5 ng/μl of trypsin, Sigma, USA) at 37°C. Trypsin was inactivated by incubating peptide samples in 0.1% formic acid (1.0 μl) for 40 min at 37°C. The digested peptides were extracted using extraction buffer (50% trifluoroacetic acid/50% acetonitrile) in an ultrasonic water bath at RT. Extracted peptides were mixed with MALDI matrix (0.5 μl) α-cyano-4-hydroxycinnamic acid (20 mg/ml) in 0.1% trifluoroacetic/30% (v/v) acetonitrile (1:2) and dried at RT followed by mass spectrometric analysis by MALDI-TOF/TOF-MS/MS using an UltrafleXtremeTM mass spectrometer (Bruker Daltonics Inc., Billerica, Massachusetts, USA). The instrument was set in positive ionization mode and was previously calibrated with a mass standard starter kit and standard tryptic BSA digest (Bruker). Proteins were identified using Mascot Software version 3.5 (https://matrixxscience.com) (Matrix Science, London, UK) at 1.2 Da MS/MS mass tolerance, in which SwissProt database was searched among Proteobacteria and one missed cleavage was allowed. Oxidation of methionine and carbamidomethylation of cysteine were considered variable and fixed modifications, respectively. Proteins identified from Mascot were functionally annotated by the UniProt database (https://www.uniprot.org).

### Label-Free Quantification

Label-free relative quantification was performed according to the method described by Sharma et al. (2014) to identify and analyze the differentially expressed proteins at a low temperature. The total protein of *Iodobacter* sp. grown at different temperatures was quantified by the Bradford method. Total protein (200 μg) from each sample was lyophilized and resuspended in 100 μl of ammonium bicarbonate (100 mM). Protein suspension was reduced with 10 mM dithiothreitol and alkylated with iodoacetamide (55 mM), followed by trypsinization (Promega, Madison, Wisconsin, USA) for 12–16 h at 37°C. Trypsin was inactivated by incubating the sample with 0.1% formic acid (1 μl) for 40 min at 37°C. Digested peptides thus obtained were lyophilized and dissolved in 0.1% formic acid. The resuspended sample (10 μl) was loaded on an Advance Bio Peptide column (2.1, 100 mm, 2.7 μm) (Agilent Technologies, Santa Clara, California, USA) equilibrated with 0.1% (v/v) formic acid (Solvent A) for 3 min with a flow rate of 5 μl/min. For peptide separation, a linear gradient starting from 5 to 45% of solvent B (0.1%, v/v) formic acid (in acetonitrile) for 75 min was employed. Three replicates per sample were used for all data acquisition using 6550 UHPLC-Q-TOF-IMS (Agilent Technologies, Santa Clara, California, USA). The following parameters were set in the acquisition mode; MS minimum range (m/z): 50, MS maximum range (m/z): 1,700, and MS and MS/MS scan rate (spectra/sec): 3. The following parameters were fixed in precursor selection; maximum precursor selection per cycle: 3, threshold (absolute): 5,000, threshold (relative %): 0.010, and target (counts/spectrum): 25,000. The instrument was set in positive ionization mode. For protein identification by Spectrum Mill software (Agilent Technologies, Santa Clara, USA), the following search parameters were used; NCBI database (version 2010_09), having 16,365 protein sequences; fixed modification was carbamidomethylation; N-terminal acetylation, N and Q deamidation, and M oxidation, as variable modifications; missed cleavage: 1 and fragment error tolerance: 0.5 Da. Accession numbers retrieved from Spectrum Mill software were annotated using NCBI. The proteins identified using label-free quantification and MALDI-TOF/TOF-MS/MS were analyzed with BLAST2GO analysis and the UniProt database, and the matched protein GIs were functionally categorized.

### Ice Recrystallization Inhibition Activity

The IRI activity of lysate was analyzed using a splat assay following Knight and Duman (1986), with slight modifications as mentioned by Kashyap et al. (2020). Total protein (1.0 mg/ml) in 30% sucrose was sandwiched in two coverslips and was flash-frozen at −20°C. It was allowed to recrystallize at −9°C for 40 min using a nanolitre osmometer (Otago Instruments, Dunedin, New Zealand). The growth of ice crystals was monitored under an upright Eclipse Ci Microscope (Nikon, Tokyo, Japan) at 10X resolution. BSA (2.0 mg/ml) was used as a negative control. Heat denaturation of crude lysate at 100°C for 10 min was performed to confirm that the activity was because of the protein content. The area of crystals was quantified using ImageJ software.

### PHA Production Under Different Physiological Conditions

In the PHA production medium, PHA production by *Iodobacter* sp. PCH194 was assessed (g/l; K_2_HPO_4_ 6.0, KH_2_PO_4_ 3.0, NH_4_Cl 0.5, NaCl 1.0, and 1.0 mM MgSO_4_, 0.1 mM CaCl_2_) under different environmental conditions, *viz.*, temperature, aeration, and pH. The bacterium was also tested to synthesize PHA at varying nutritional conditions of carbon to nitrogen ratio from 0.2 to 60 (where NH_4_Cl concentration was kept constant at 0.05% and glucose concentration varied accordingly). Biomass and PHA production were quantified as described by us in earlier studies (Kumar et al., 2018, 2020a).

### Morphology and Anatomy of *Iodobacter* sp. PCH194

The cell morphology of bacterial cells from the late log phase was examined using TEM analysis using JEOL JEM-1010 (JEOL, Akishima, Japan). The PHA granules formation by bacterial isolate was analyzed as per the methods described by Sandoval et al. (2007) with slight modification. The bacterial cells were grown to stationary phase in 2.0% glucose and harvested at 2,400 *g*, washed three times with phosphate buffer saline, and fixed by glutaraldehyde solution (2.0%, v/v) at 4°C. Samples were washed three times with PBS buffer and fixed with 1.0% osmium tetroxide for 60 min in the dark. The washing step was repeated, stained in blocks with uranyl acetate (30%), and embedded slowly in Embed 812 epoxy resin (Electron Microscopy Sciences, Hatfield, Pennsylvania, USA). Polymerization of blocks was carried out in an oven at 50°C for 72 h. Ultrathin sections of the samples were cut using an ultramicrotome, and further grids were prepared. Structural studies were performed with an FEI Technai G2200kV transmission electron microscope (FEI, Hillsboro, Oregon, USA).

### Synthesis of Violacein Pigment by *Iodobacter* sp. PCH194 and Its UV Protective Role

Violacein was produced by *Iodobacter* sp. PCH194 growing in glucose and tryptone medium, and extracted as described by Kumar et al. (2021). For analyzing the protective role of violacein pigment against UV, the cells of *Iodobacter* sp. PCH194 were drawn at a different time interval at the initial log phase (when cells are non-pigmented), late log phase, and stationary phase (when cells produce pigments), washed in normal saline and diluted to 1.0 OD_600_. Different dilutions were spread on a nutrient agar plate and treated with 302 nm UV radiation (UV B_302_) from a distance of 1.4 feet by using UV torch (Analytik Jena, California, USA) for 1, 2, and 3 min. The dose of UV-B was 0.32 Js^−1^cm^−2^ (320 mWcm^−2^) as measured by a UV light meter (Model UV340A, Lutron Electronic, Pennsylvania, USA). UV-untreated plates from each growth phase served as controls. Non-pigmented cells of *Iodobacter* sp. PCH194 were harvested after 16 h of growth, amended with methanol-extracted violacein pigment (100 μg/ml), and compared to non-pigmented cells without violacein in a UV exposure experiment as described. All plates were further incubated at 20°C for 2–3 days, and the number of colonies was counted and recorded as log cfu/ml. The significance of the experiment was determined by the *t*-test, and the level of significance was expressed as *P*-value at ^*^ ≤ 0.05, ^**^ ≤ 0.01, and ^***^ ≤ 0.001.

## Results

### Sampling Site and Bacterium Description

**Figure 1:**
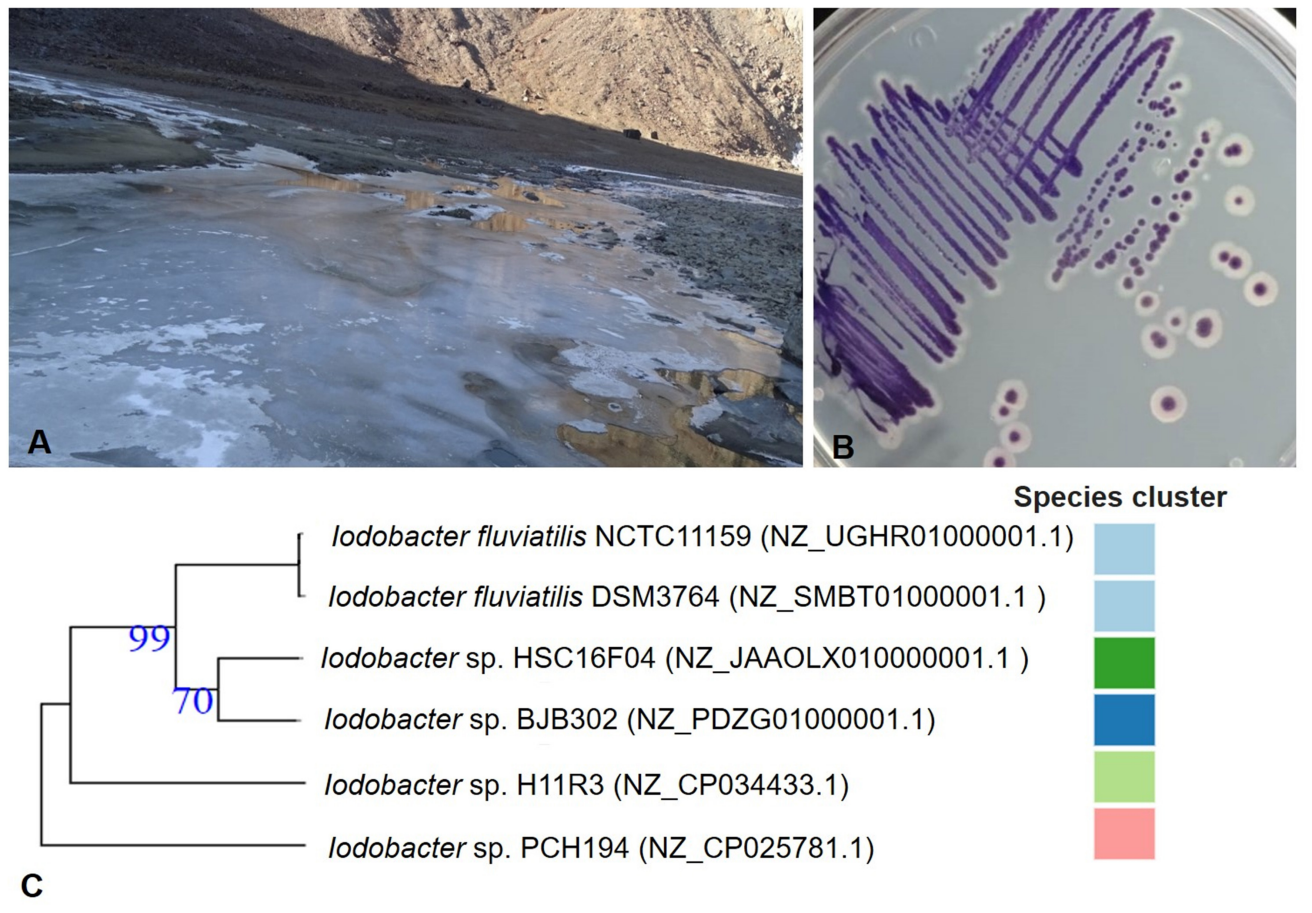
Sampling site and Himalayan bacterium *Iodobacte*r sp. PCH194. **(A)** A view of partially frozen kettle lake (Picture was taken on 18 November 2016) at an altitude of 4,160 masl and geographical coordinates of 33.006831°N, 76.248209°E at Sach Pass area in Chamba district of Himachal Pradesh, India, **(B)** isolated single colonies of *Iodobacter* sp. PCH194, and **(C)** a phylogenetic tree for genus *Iodobacter* based on the available whole-genome sequences. The number in parenthesis is the NCBI reference number for the respective genome assembly, and the color code represents the species cluster, where different colors represent the different species. The tree was constructed using an online TYGS server.

The Bhoot ground lake at Sach Pass (Pangi Valley, Himachal Pradesh, India) is a shallow kettle lake formed due to the glacier retreat and remains frozen for almost half of the year (Figure 1A). The lake water temperature, pH, and UV radiation were 4°C, 4.5, and 50 mWcm^−2^, respectively; measured at the site during the sampling (18 November 2016). Available N, P, K (kg/Ha), electrical conductivity (μS/cm), and pH of dried mud vary from 65.86 to 194.9, 11.68 to 25.76, 68.26 to 162.13, 88 to 95, and 4.1 to 4.26, respectively (Supplementary Table 1). The isolated bacterium *Iodobacter* sp. PCH194 forms violet-pigmented colonies (Figure 1B). The cells were found to be in straight round-ended rods, Gram-negative, facultative anaerobes, oxidase and catalase-positive, and glucose-fermenting. The bacterium can grow at −5 to 25°C, with optimum growth at 20°C (Supplementary Table 2).

### General Description of the Genome and Phylogenomic Analysis

Whole-genome sequencing of *Iodobacter* sp. PCH194 has resulted in a single high-quality contig of a 4.44 Mb chromosome and three sizes of 76.7, 57.4, and 12.3 kb plasmids. The NCBI predicted 4,165 genes, 3,950 proteins, 31 rRNA, 76 tRNA, 04 ncRNAs, and 104 pseudogenes (Table 1). The phylogenomic analysis of *Iodobacter* sp. PCH194 with reference strains *I. fluviatilis* DSM 3764 and *I. fluviatilis* NCTC11159 showed a low value of ANI (80.81%) and DDH (25.90% and 26.40%). Similarly, other strains of *Iodobacter*, i.e., BJB302, H11R3, and HSC-16F04, also showed a low percentage in ANI and DDH analysis (Supplementary Table 3). The phylogenetic tree was constructed using the TYGS server based on the available whole-genome sequences in the NCBI database. *Iodobacter* sp. PCH194 showed a separate and distinct clade compared to the rest of the *Iodobacter* spp. (Figure 1C). The low ANI (79.99–80.81), DDH (25.70–26.40) score, and phylogenetic analysis of strain PCH194 clearly describe it as a putative novel species of the genus *Iodobacter*.

**Table 1:** Genomic features of *Iodobacter* sp. PCH194.

**Attributes**	**Value**				
	**Genome**	**Chromosome**	**Plasmid 1**	**Plasmid 2**	**Plasmid 3**
RefSeq	–	NZ_CP025781.1	NZ_CP025783.1	NZ_CP025782.1	NZ_CP025784.1
Genome size (bases)	4,588,033	4,441,511	76,766	57,428	12,328
GC content (%)	47.50	47.50	50.36	40.90	51.21
Proteins	3,950	3,797	67	70	16
rRNAs	31	31	0	0	0
tRNAs	76	76	0	0	0
other RNAs	04	04	0	0	0
Genes	4,165	4,006	72	71	16
Pseudogenes	104	98	05	01	0

### Genomic Insights for Adaptive Strategies of *Iodobacter* sp. PCH194

Genomic analysis of *Iodobacter* sp. PCH194 revealed multiple genes/proteins for stress adaptation (Table 2). The adaptive genomic traits are described in detail in the following subheads and are tabled in Supplementary Data Set 1.

**Table 2:** Adaptive genomic traits of *Iodobacter* sp. PCH194. Adaptive strategies to stress conditions and related genes/proteins are tabulated along with their putative physiological and ecological functions.

**Stress conditions**	**Adaptation strategies**	**Genes/proteins for adaptative** **strategies**	**Physiological functions**
Low temperature	Cold stress response	*dnaJK, groES, groEL, hscAB, surA,* *hslO, clpB, betIAB* adhesins, AFP	Growth at low temperature, protection from freezing
Freezing	Antifreeze proteins		
Oxidative stress	Oxidative stress response	Superoxide dismutase, catalase, peroxidases, thioredoxin, peroxiredoxin	Prevention and detoxification from oxidative damage
Nutritional fluctuation and starvation	Chemotaxis and flagellar motility	*cheA,W,R,Y,D* *motABCD, flaABH, fliMNOPQRS,* *fliEFGI, flgBCDEFGHIJKL*	Sensing the nutrition and movement
	Starvation response proteins	ppGpp synthetase, *clpXP, psiF*	Starvation sensing, signaling and response
	Carbon storage	*phaC, phaB, phaA, phaR, phaZ,* *phaJ*, phasin protein	Carbon storage and energy reservoir
Low oxygen	Harvesting low oxygen and facultative anaerobic respiration	High affinity terminal oxidases (*Cbb*_3_ and *bd*) *fnr, napABCF, frdABCD, arsBC J*	Respiration in low O_2_ or anaerobic condition
UV irradiance	DNA repair, violacein production	*UvrABC*, photolyase *vioABCDE*	UV protection and tolerance
Predation and competitive stress	Biofilm and violacein pigment synthesis	*luxS, Q*, Enzymes of GT family, *vioABCDE*	Biofilm formation, antimicrobial and anti-predatory
Acidic pH	Efflux pump, Na^+^/H^+^ antiporters, amino acid dependent acid tolerance	*kdpFABC, nhaCR*, trkAD*, arcD*, ADI, OTC, CK, GAD	Removal, detoxification and consumption of H^+^
Overall physio- chemical/environment stress	Toxin-antitoxin system, plasmids	Toxin-antitoxin system, genes for conjugation assembly proteins	Cell persistence, recombination, and adaptative role

### Starvation and Storage of Carbon

The genome of *Iodobacter* sp. PCH194 showed the presence of key genes involved in nutritional starvation, viz., carbon sensing and downstream response mechanisms such as carbon starvation protein A (*cstA*), sigma factor (*rpoS*), stringent starvation proteins A and B (*sspAB*), bifunctional ppGpp synthetase, Clp proteases (*clpA,B,S,X,P*), and phosphate starvation-inducible protein (*psiF*). The genes for polyhydroxyalkanoate (PHA) synthesis, namely, polyhydroxyalkanoate synthase (*phaC*) and acetyl-3-hydroxybutyryl-CoA dehydrogenase (*phaB*), were found in two copies each, while acetyl-CoA-acyltransferase or thiolase (*phaA*) was found in a single copy. Additionally, one copy of PHA depolymerase (*phaZ*) and PHA regulatory gene *(phaR)*, two genes encoding for phasin family protein, and three copies of enoyl CoA (*fadB* or *phaJ*) were also present.

### Respiration

The genomic insights revealed the presence of genes for various high-affinity terminal oxidases, *viz*., cytochrome *cbb*_3_ and cytochrome *bd*, and an operon of NADH-quinone oxidoreductase (*nuoABCEFGHIJKLMN*). The genome analysis also showed various genes encoding for fumarate/nitrate reduction transcriptional regulator Fnr (*fnr*), two-component system response regulator NarL (*narL*), and various reductases such as periplasmic nitrate reductase (*napABCF*), fumarate reductase (*frdABCD*), and arsenate reductase (*arsBCJ*).

### Genomic Features for Cold, UV, Oxidative, and Osmotic Stress

NCBI annotation revealed genes for cold stress, i.e., cold-shock proteins, molecular chaperones, co-chaperone, chaperonin, fatty acid desaturase, and stress proteins. The genes encoding for the proteins involved in oxidative stress include superoxide dismutase, catalase, peroxiredoxin, thioredoxin, and various peroxidases, and the excinuclease repair system (*uvrABCD*) were also present in the genome. Additionally, genes encoding for choline ABC transporters and permease, betaine aldehyde dehydrogenase, and choline dehydrogenase involved in osmolyte glycine-betaine synthesis were present.

### Violacein Biosynthesis and Biofilm Formation

A complete operon containing five genes, *vioABCDE* encoding enzymes involved in violacein pigment biosynthesis and quorum sensing regulatory genes (*luxS* and *luxQ*), was present in the genome. The bacterium possesses genes (*epsDEF GI*) for the biosynthesis of polysaccharides and export. Furthermore, the dbCAN meta server found genes encoding for 13 different glycosyltransferase (GT) family proteins in the genome.

### Additional Adaptive Features

The genome of *Iodobacter* sp. PCH194 possesses key genes for chemotaxis (*cheA, D, W, R, Y*) and flagellar assembly (*motABCD, fliMNOPQRS, fliEFGI, flaABH, flgBCDEFGHIJKL*). The genome showed the presence of genes encoding for the cation-efflux pump, cation transporters and Na^+^/H^+^ (sodium:proton) antiporters, potassium channel protein, potassium transporter (Kup), and an operon *kdpFABC* encoding for the ATP-dependent potassium transporter system. The bacterium also possesses the genes encoding for the enzymes involved in amino acid-dependent acid tolerance, i.e., arginine-ornithine antiporter (ArcD), arginine deiminase (ADI), ornithine carbamoyltransferase (OTC) and carbamate kinase (CK), glutaminase, glutamate decarboxylase, lysine decarboxylase, and histidine decarboxylase. Apart from other mentioned strategies, the genome of *Iodobacter* sp. PCH194 possesses genes encoding for the toxin-antitoxin (TA) system on chromosomes and plasmid. Additionally, three plasmids possess various genes encoding for replication proteins, transposase, recombinase, replicase, mobilization protein, conjugal transfer protein TraA, and transcriptional regulators.

### Proteomic Response of *Iodobacter* sp. PCH194 to Cold and Freezing Conditions

#### Growth Kinetics and General Proteome Response to Cold and Freeze

**Figure 2:**
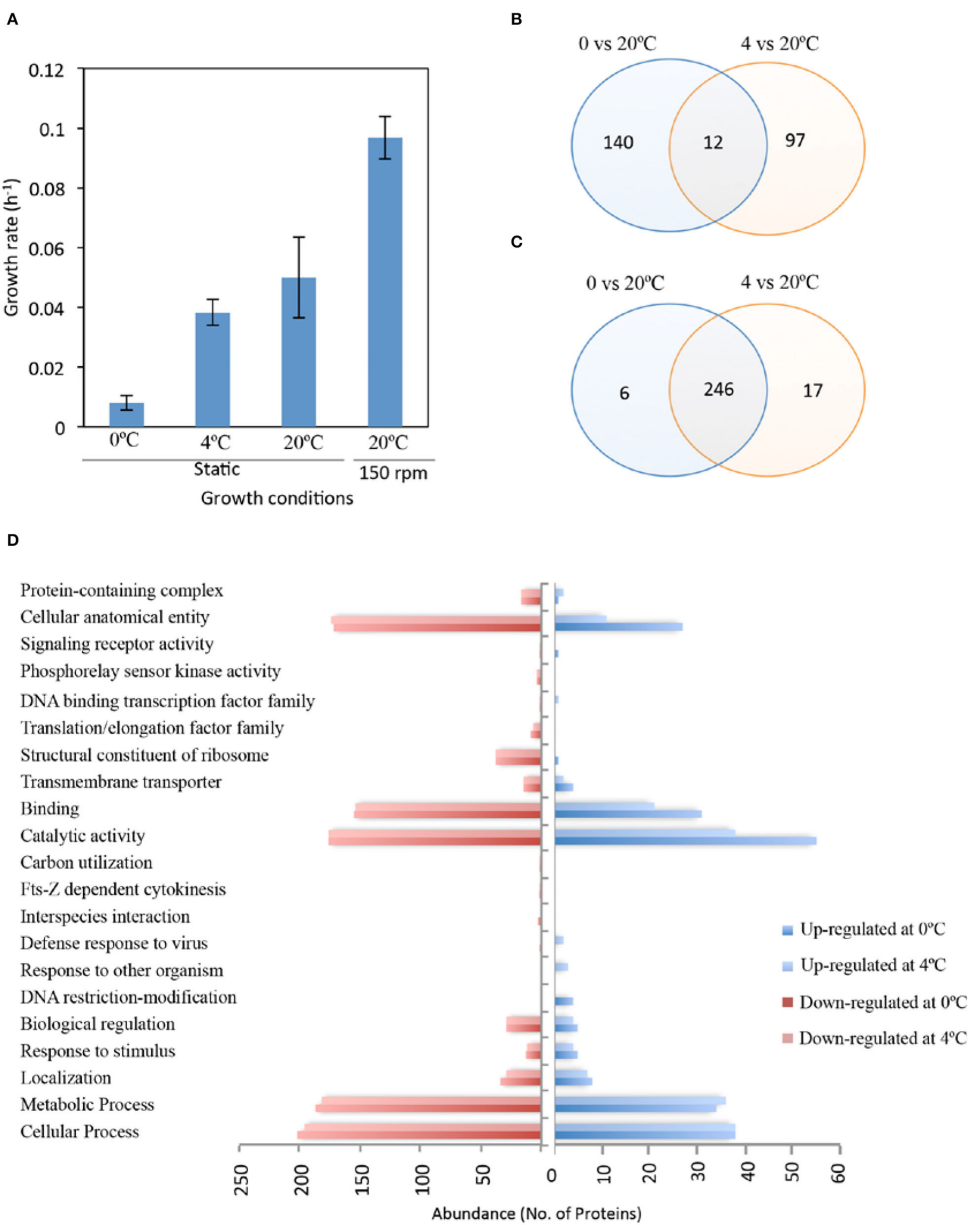
Growth and proteomic response of *Iodobacter* sp. PCH194 in cold/freeze conditions. **(A)** Growth rate (OD_460_) of *Iodobacter* sp. PCH194 at different temperatures in nutrient broth medium, **(B)** Venn diagram showing the total and shared number of upregulated, and **(C)** downregulated protein targets in response to cold (4°C) and freeze (0°C) compared to 20°C identified by gel-based and gel-free proteomics approaches, and **(D)** the abundance of upregulated and downregulated proteins with functional categorization by UniProt.

*Iodobacter* sp. PCH194 grew at a temperature ranging from −5 to 25°C, with optimum growth at 20°C. The growth rate (μ) of the bacterium calculated in NB medium (pH: 7.0) at 0, 4, and 20°C in static, and at 20°C with 150 rpm was 0.008, 0.038, 0.049, and 0.096 h^−1^, respectively (Figure 2A). The bacterium under cold stress (at 4 and 0°C conditions) underwent proteome changes as visualized using SDS-PAGE (Supplementary Figure 1). A total of 19 differentially expressed polypeptides were observed and subjected to mass spectrometric identification using MALDI-TOF/TOF. After Mascot analysis, 68 upregulated and 17 downregulated protein targets were identified (Supplementary Data Set 2).

In contrast, the label-free proteomics data led to the quantitative identification of 558 proteins (p < 0.05), showing a ≥1.5-fold increase and ≤ 0.75 decrease in abundance upon cold/freeze stress (Supplementary Data Set 3). Based on the gel and gel-free approaches, 152 and 109 proteins were upregulated at 0 and 4°C, respectively, compared to 20°C (Figure 2B). Among all proteins that were upregulated at cold temperatures, 125 and 64 proteins were identified exclusively at 0 and 4°C, respectively. The bacterium also revealed downregulation of 252 and 263 proteins at 0 and 4°C, respectively (Figure 2C). These downregulated proteins include the majority of proteins expressed at 20°C. The gene ontology (GO) study of differently expressed proteins under cold (4°C) and freezing (0°C) conditions revealed upregulated and downregulated targets in the cellular component and biological process category (Supplementary Figure 2). Cellular components that were upregulated at 0°C as compared to 4°C included a higher number of membrane proteins and intrinsic components of membrane proteins. Other proteins belonging to these categories were found to be downregulated in both cold and freezing conditions. These results indicate an important role for membrane dynamics during freeze stress. The upregulated and downregulated protein targets were further functionally categorized using the UniProt database (Figure 2D, Supplementary Data Set 4). Upon cold stress, the bacterium showed differential regulation in cellular and metabolic processes such as nucleotide biosynthesis, transcription, translation, DNA recombination and repair, peptidoglycan, and fatty acid metabolism. Additionally, proteins involved in maintaining the structural and functional integrity of cells (cellular anatomical entity) and transmembrane transporters also exhibit different protein regulations during low temperatures. Since these categories show differential proteome responses, their functions in *Iodobacter* sp. PCH194 were further analyzed to reveal cold-adaptive strategies.

#### Differential Proteome Response for Important Functional Categories to Cold and Freeze

The proteome changes in *Iodobacter* sp. PCH194 in response to cold/freezing stress were functionally categorized (Supplementary Table 4). Cold and freeze stress cause misfolding of proteins. Therefore, chaperones and proteases play a crucial role during cold/freeze stress conditions. *Iodobacter* sp. PCH194 showed an abundance of molecular chaperones DnaJ and HscA at freezing temperatures. However, chaperone GroEL and co-chaperone GrpE exhibited a decreased abundance, while chaperone DnaK was slightly reduced during cold stress. Additionally, ATP-dependent Clp proteases were also found to be upregulated in both gel-based and gel-free studies during low-temperature stress. Cold/freeze stress-regulated not only chaperones and proteases but also RNA metabolism, mediated by cold shock proteins (CSPs) and ribonucleases (RNase). RNase E and RNase Z were found abundant at 0°C in *Iodobacter* sp. PCH194, whereas RNase II and RNase BN were not detected at low-temperature conditions.

Interestingly, the proteins of secretion system VI and IV families were found to be upregulated during cold/freeze stress. In addition, T4SS and T6SS, phage tail proteins closely resembling T6SS, were also upregulated at freezing temperature (0°C). Besides, the glycosyltransferase (family 1) protein that participated in biofilm formation was also upregulated during low temperatures. One of the strategies to survive under subzero temperatures is the production of antifreeze proteins such as adhesins. In *Iodobacter* sp. PCH194, adhesin was detected in all three temperatures, but its expression decreased during 4°C stress and then increased at 0°C. Furthermore, MafB adhesion was detected only at 0°C, indicating that it might also have a role as an antifreeze protein during freezing conditions.

#### Ice Recrystallization Inhibition Activity in *Iodobacter* sp. PCH194

**Figure 3:**
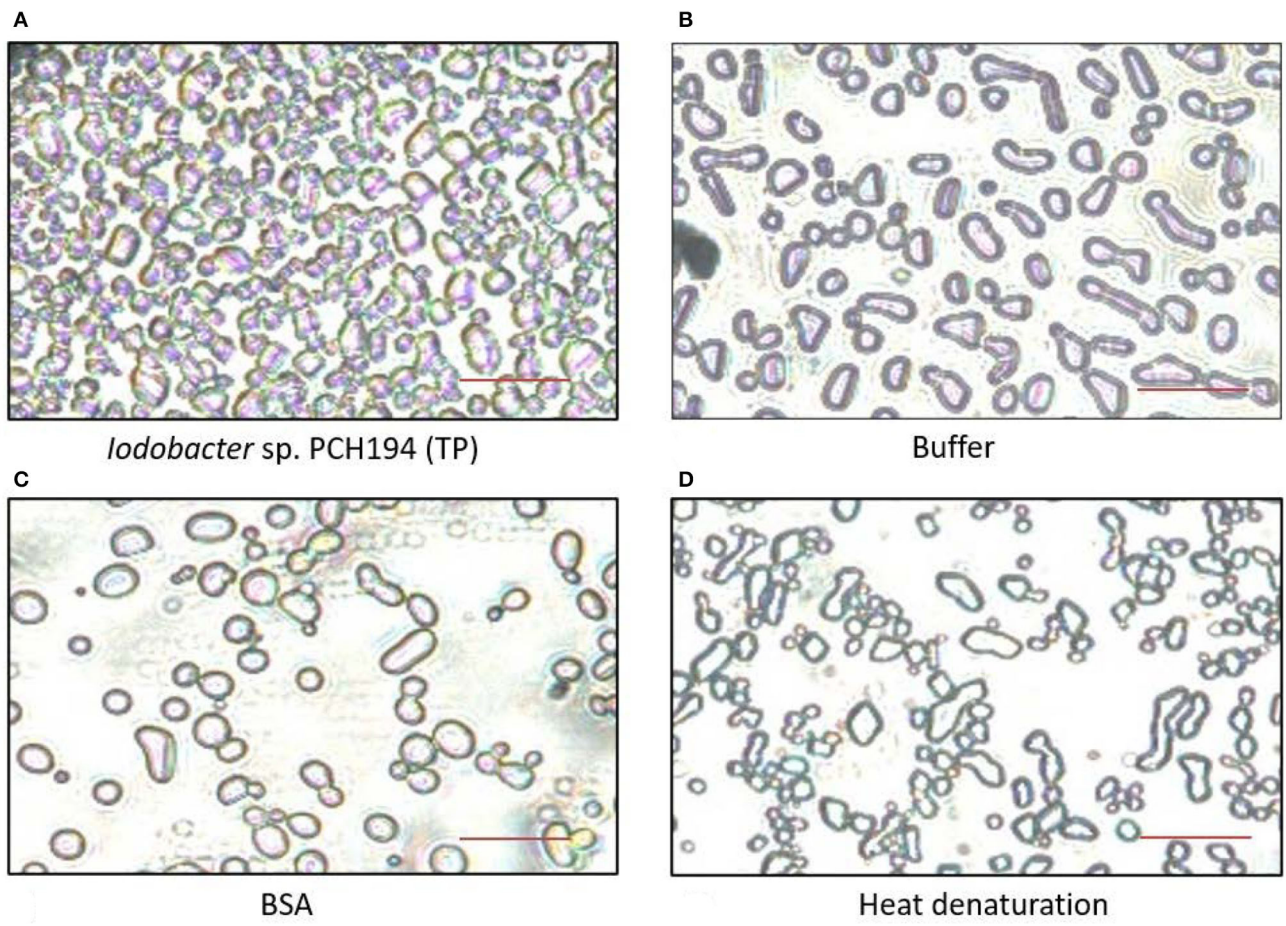
Antifreeze activity exhibited by *Iodobacter* sp. PCH194. The ice recrystallization inhibition (IRI) was observed at −9°C after 40 min. **(A)** *Iodobacter* sp. PCH194 [total cell lysate protein (TP), 1.0 mg/ml] showed smaller ice crystals upon recrystallization in comparison to **(B)** buffer (20 mM Tris-HCl, pH 8.0), and **(C)** BSA (1.0 mg/ml) as a negative control, and **(D)** heat denaturation (at 100°C for 10 min) of *Iodobacter* sp. PCH194 (TP) resulted in the loss of activity. Scale bar = 200 μm.

Antifreeze activity in crude lysate proteins (1.0 mg/ml) of *Iodobacter* sp. PCH194 was validated by inhibition of ice recrystallization at −9°C, when *Iodobacter* sp. PCH194 lysate decreased the average area of ice crystals by a factor of 4 compared to BSA (1.0 mg/ml) as a negative control. Heat denaturation of the protein lysate resulted in the loss of the IRI activity and produced a 5-fold increase in the average area of ice crystals (Figure 3). The IRI activity in crude lysate indicated that antifreeze proteins are constitutively present in *Iodobacter* sp. PCH194.

### Physiological Insights for Adaptive Strategies of *Iodobacter* sp. PCH194

#### Carbon Storage Under Different Physiological Conditions

**Figure 4:**
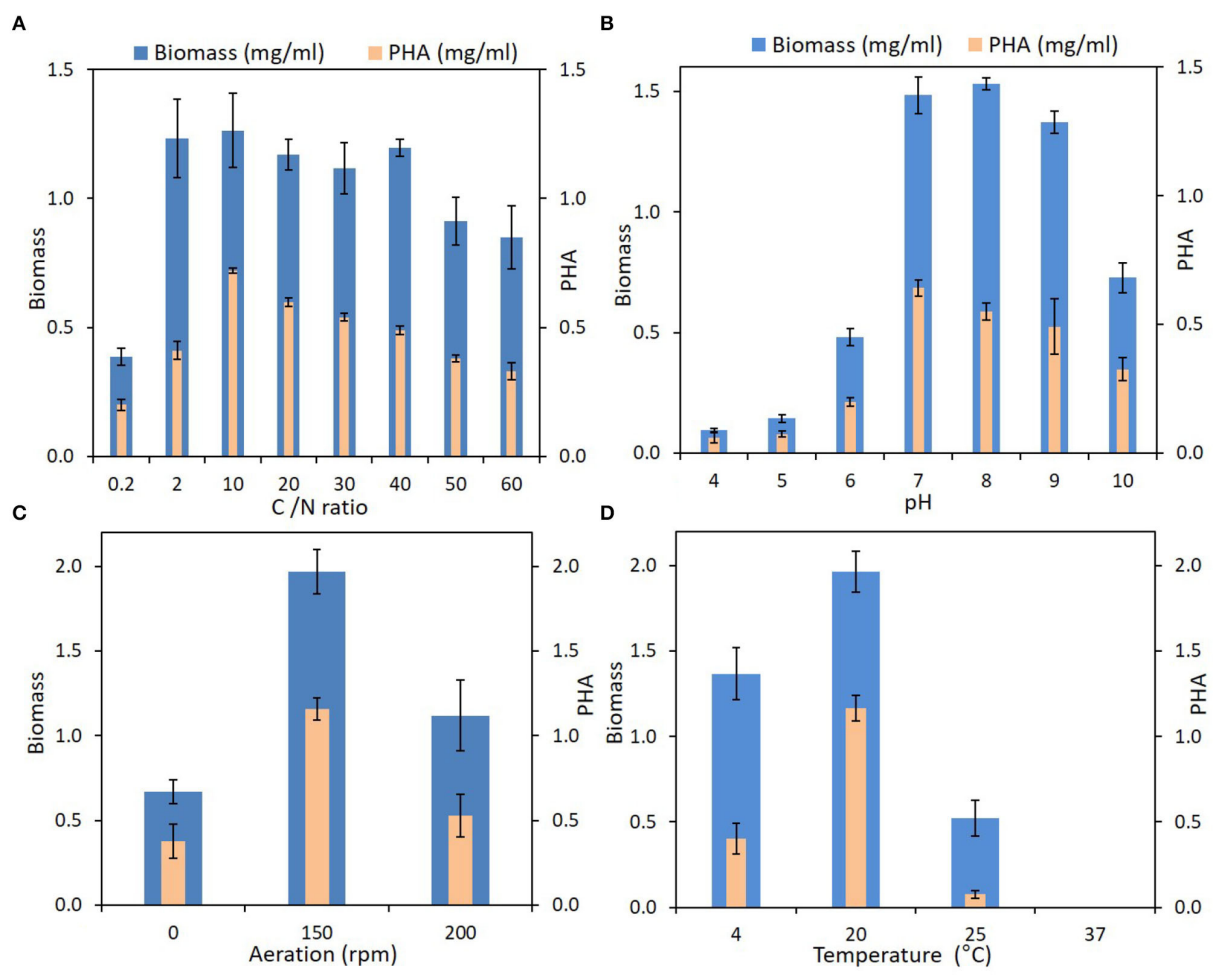
PHA production by *Iodobacter* sp. PCH194 at different physicochemical and environmental conditions. **(A)** Carbon to nitrogen (g/g) ratio, **(B)** pH, **(C)** aeration, and **(D)** temperature.

*Iodobacter* sp. PCH194 showed the ability to synthesize PHB in different conditions of pH (4–10), temperature (4–25°C), varied carbon to nitrogen ratio, and in static as well as shaking conditions (Figure 4). The bacterium synthesizes 40–60% PHB to its dry cell mass in a wide range of carbon to nitrogen ratios of 0.2–60 (glucose and NH_4_Cl as sole carbon and nitrogen sources). Though the growth is slow in the medium with a C/N ratio of 0.2, the bacterium can synthesize 40% of PHB to its dry cell mass. The TEM analysis of an ultrathin section of the stationary phase showed the formation of intercellular PHB granules.

#### Violacein Pigment Production and Formation of Biofilm

**Figure 5:**
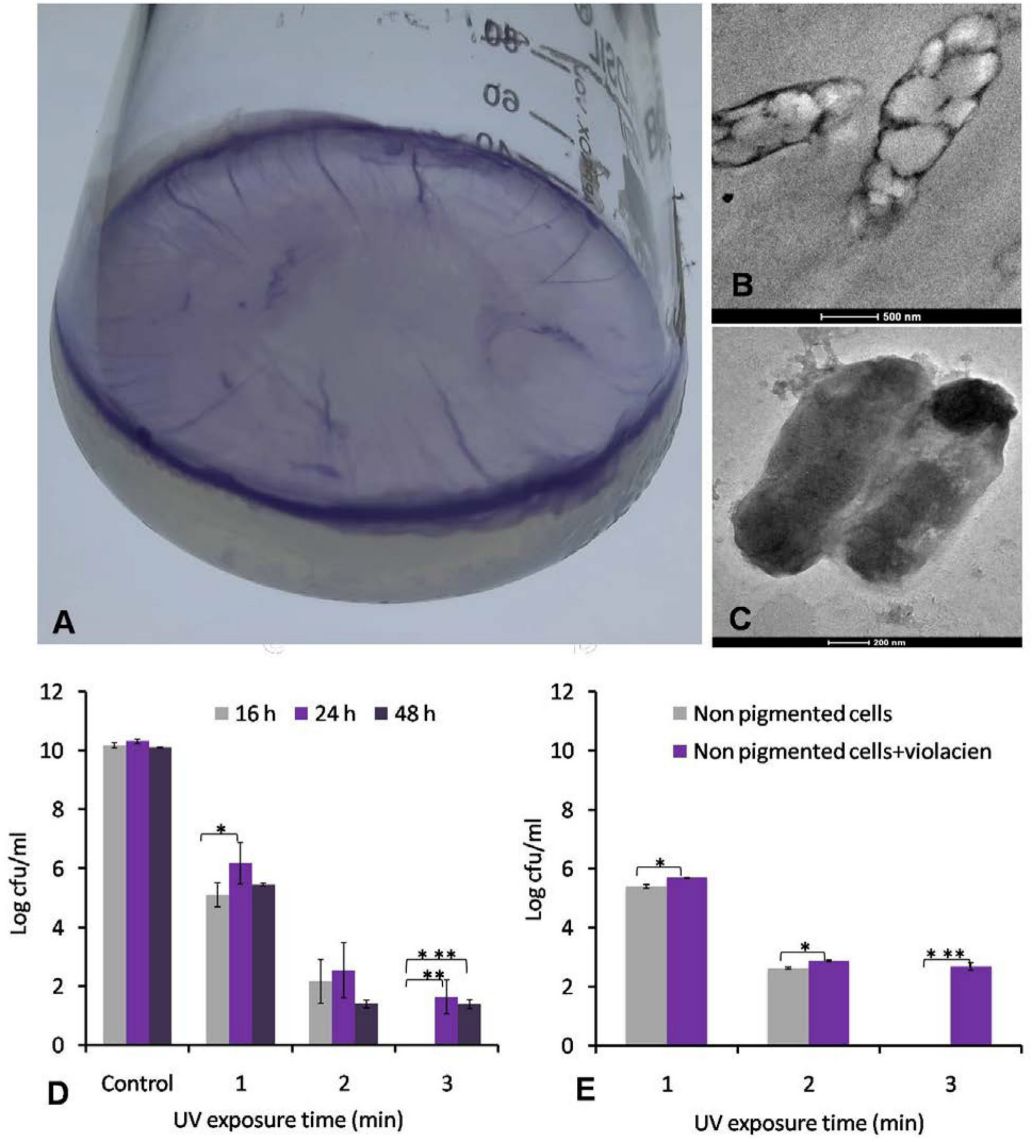
Formation of violate biofilm by *Iodobacter* sp. PCH194 and UV protective role of violacein pigment. **(A)** Formation of violet-colored mat on the surface of medium when growing at 4°C under static conditions, **(B)** TEM image of ultrathin sections of cells showing the presence of PHA granules, **(C)** morphology of bacterial cells showing the presence of a sheath around the cell, **(D)** effect of UV irradiance to cells of *Iodobacter* sp. PCH194 harvested at different growth phases, *viz*., non-pigmented cells of the initial log phase and pigmented cells of exponential log phase and stationary phase (expressing violacein pigment 43–50 and 41–45 μg/ml/OD_460_), and **(E)** effect of violacein (100 μg/ml) addition to UV tolerance of non-pigmented cells. The level of significance was expressed as a *P*-value as * ≤ 0.05, ** ≤ 0.01, and *** ≤ 0.001.

Physiological experiments showed that *Iodobacter* sp. PCH194 can grow and produce violacein pigment under static and shaking conditions in the NB medium. The cells adhered to the media's surface under static conditions and formed a violet-colored mat in 7–8 days of incubation at 4°C (Figure 5A). The bacterium also formed a violet-colored biofilm around cells under shaking conditions as well. The TEM analysis of stationary phase bacteria grown in NB at 20°C and 150 rpm showed intercellular PHB granules and a clear sheath-like structure around the cells (Figures 5B,C).

#### UV Protective Role of Violacein Pigment

The colonies of *Iodobacter* sp. PCH194 are violet pigmented, and they also produce violet pigment in a liquid medium. During undisturbed growth at 4°C, the bacterium produces a violet-colored mat at the medium's surface (Figure 5A). Violacein is likely to protect bacterial cells against UV irradiation at high altitudes. Therefore, to validate the role of violacein pigment against UV stress, the non-pigmented and pigmented cells of *Iodobacter* sp. PCH194 were subjected to a UVB irradiance of 320 mWcm^−2^. The effect of UV irradiance on the bacterial cells from different growth stages showed that the pigmented cells have higher tolerance and survival under UV. Pigmented cells from the late log phase showed 21% higher survival at 1 and 2 min and 10% higher survival at 3 min of UV exposure. Similarly, pigmented cells from the stationary phase showed 13%, 7%, and 6% higher survival than the non-pigmented cells in 1, 2, and 3 min of UV exposure (Figure 5D). The violacein pigment treated (100 μg/ml) non-pigmented cells of *Iodobacter* sp. PCH194 showed better UV tolerance than untreated cells (Figure 5E).

## Discussion

**Figure 6:**
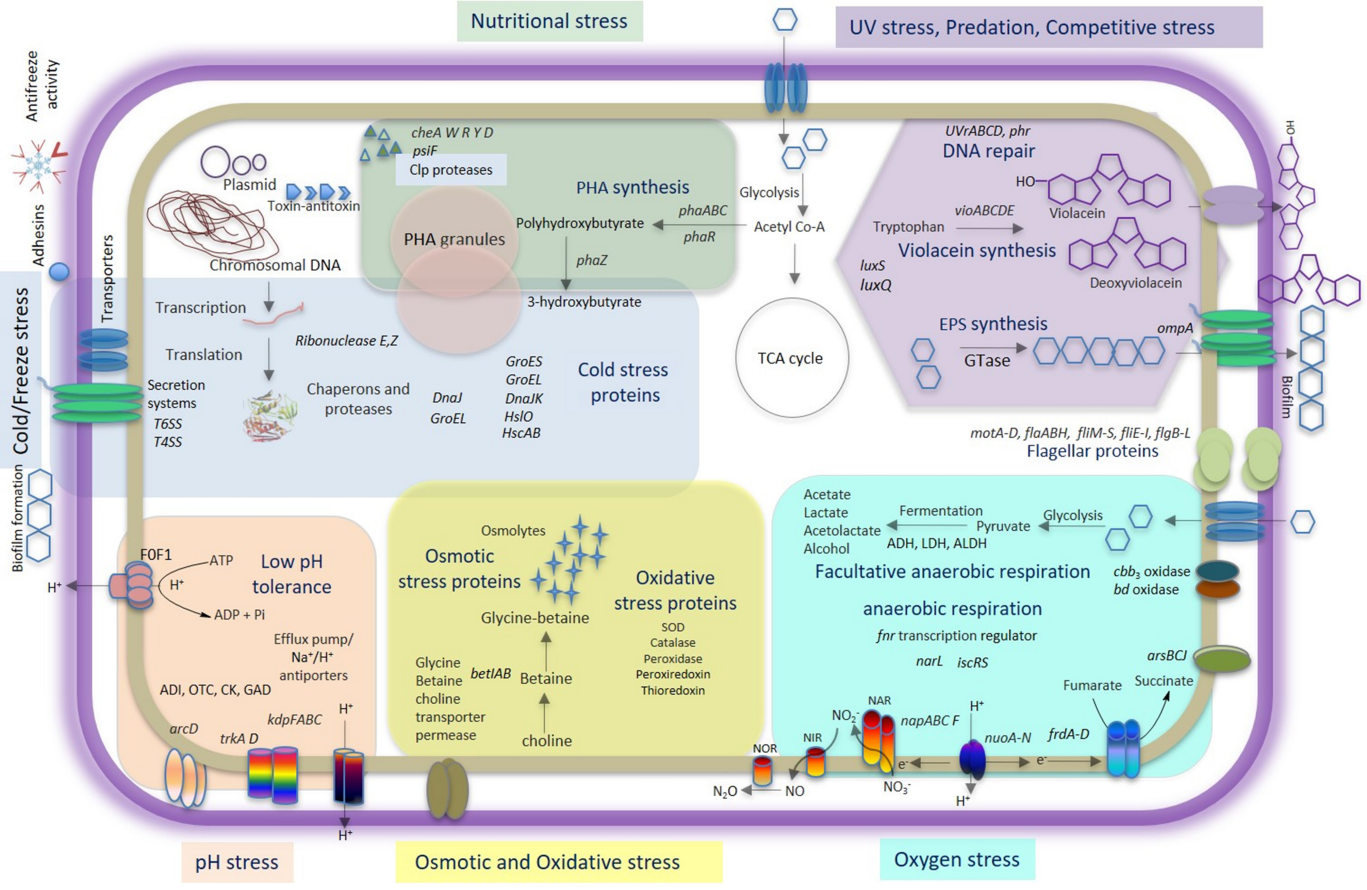
Comprehensive model of *Iodobacter* sp. PCH194 adaptive strategies. The model is based on genomic, proteomic, and physiological data showing multiple strategies of *Iodobacter* sp. PCH194 to adapt to the predominantly frozen kettle lake environment in the high-altitude Himalayas.

This study unveiled multiple adaptive strategies of a unique Himalayan bacterium, *Iodobacter* sp. PCH194, ensuring its survival in high-altitude stresses (Figure 6). It also provides a complete genome sequence of Himalayan *Iodobacter* and highlights the adaptive traits in its genome. Furthermore, differential proteome response to cold and physiological experiments of antifreeze activity, PHB, and violacein synthesis supports its survival in the stress environment of high-altitude Himalayas. Additionally, the phylogenomic analysis suggested that *Iodobacter* sp. PCH194 is a putative novel bacterium in the genus *Iodobacter* with biotechnological importance. Herein, we discuss multiple adaptive strategies of *Iodobacter* sp. PCH194, supported by its genomic, proteomic, and physiological data.

Cold stress is one of the major environmental stresses in the high-altitude Himalayan lakes. *Iodobacter* sp. PCH194 showed adaptation to changes in temperature and can grow from subzero to 25°C. Wide temperature adaptability was supported by many genes encoding for cold/freeze and other stress adaptation functions. Previous studies also reported a high copy number of genes for general stress, cold shock, osmotic, and oxidative stress in bacteria from the Antarctic and Arctic cryo-environments (Mykytczuk et al., 2013; Goordial et al., 2016). In response to the cold, *Iodobacter* sp. PCH194 revealed a differential proteome expression. During cold stress, bacteria reduce cell growth, and there is a downshift in the efficiency of transcription and translational machinery with an increase in the Csps proteins (Polissi et al., 2003). The proteins that show the most significant changes upon cold stress have been known to be involved in nucleotide biosynthesis, translation processes, DNA recombination and repair, peptidoglycan, and fatty acid metabolism (De Maayer et al., 2014; Baraúna et al., 2017). Proteins regulating all these functions were differentially regulated in *Iodobacter* sp. PCH194 upon low-temperature stress. The roles of the proteins in these categories are well-established in many cold-loving bacteria, such as *Planococcus halocryophilus* (Mykytczuk et al., 2013), *Sphingopyxis alaskensis* (Ting et al., 2010), and *Psychrobacter* sp. (Koh et al., 2017).

Chaperones and proteases play an important role in the cold adaptation of bacteria. *Iodobacter* sp. PCH194 chaperone system responded to low-temperature stress by increasing the expression of DnaJ, while the expression of DnaK did not show much change. DnaJ is known to interact with unfolded proteins and prevent their aggregation (Han and Christen, 2004). Besides, it also acts as Hsp40 and interacts with Hsp70 heat shock proteins (Hennessy et al., 2005). An interesting observation during the cold stress response of *Iodobacter* sp. PCH194 was the abundance of RNase E and RNase Z at 0°C. Low-temperature stress triggers the RNase to maintain the optimum transcript levels. DEAD-box helicases are associated with RNase E-based degradosomes under cold shock conditions to degrade structured RNAs (Prud'homme-Genereux et al., 2004; Barria et al., 2013). Furthermore, the stress response regulator *rpoS* is known to be degraded by RNase E and RNase III (Basineni et al., 2009). The RNA polymerase sigma factor RpoD responsible for transcription of constitutive genes was detected in Himalayan *Iodobacter* while growing at 4°C, but RpoS was not found at cold or freeze stress. This might be because of *rpoS* degradation by upregulated expression of RNase E. Therefore, RNases seem to play an important role in the cold adaptation of *Iodobacter* sp. PCH194. In addition, upregulated T4SS and phage tail proteins closely resembling T6SS (Pukatzki et al., 2007) play a role in communication with their population, competing with others, and interacting with the environment (Russell et al., 2014).

Various organisms produce antifreeze proteins to cope up with the freezing conditions. During freezing, smaller crystals are formed in huge numbers. Their formation does not damage cells. However, large crystals harm cells during a process called recrystallization. During recrystallization, the smaller ice crystals grow by absorbing water and merging smaller ice crystals into larger ones. Antifreeze proteins (AFP) bind to ice crystals and prevent their growth during recrystallization, which results in an increased number of smaller ice crystals. Hence, AFP mitigate the damage caused to the cells. AFP are known to act extracellularly, for example, in plant apoplast space (Gupta and Deswal, 2012), while they operate in the periplasm and as adhesins in bacteria (Gilbert et al., 2005). The upregulation of adhesins in *Iodobacter* at 0°C indicated that they might play a role in freeze protection similar to MpAFP from the Antarctic bacterium *Marinomonas primoryensis* (Gilbert et al., 2005; Guo et al., 2017). The presence of IRI activity in *Iodobacter* sp. PCH194 validated the antifreeze proteins as one of the survival strategies to cope with the freezing climate of the Himalayan kettle lake. Bacterial AFP protect the cells from frost damage by inhibiting ice crystal growth during recrystallization. This study reports antifreeze activity in a Himalayan bacterium for the first time. Furthermore, antifreeze activity without cold acclimation indicates that the activity is constitutively inherited in PCH194 to cope with a frequent drop in daily temperature in the kettle lake.

Kettle lakes have frequent changes in physicochemical environments (Reverey et al., 2018); thus, nutritional fluctuation is inevitable. The presence of genes for chemotaxis and flagellar assembly in the bacterium suggested its ability to sense chemical and nutritional change and swim using flagella (Bren and Eisenbach, 2000). *I. fluviatilis, I. limnosediminis*, and *Iodobacter* sp. 7MAnt showed polar and lateral flagella (Logan, 1989; Su et al., 2013; Atalah et al., 2020). PHA synthesis to store carbon is a central strategy of many microbes in response to environmental and nutritional stress (Obruca et al., 2018; Kumar et al., 2020b). *Iodobacter* sp. PCH194 can synthesize and utilize PHA as a carbon reserve, evident from the genome analysis showing a complete set of genes for PHA synthesis (*phaABC*) and PHA depolymerization (*phaZ*). Accordingly, physiological experiments with various carbon/nitrogen ratios and environmental conditions revealed that the bacterium could synthesize PHB in the kettle lake environment. This has suggested the central role of PHB for *Iodobacter* sp. survival in the kettle lake. Apart from its role as a carbon reserve, PHA provides resistance to bacterial cells against multiple environmental stresses, including cold, freezing, oxidative, osmotic, and UV (Slaninova et al., 2018; Sedlacek et al., 2019; Obruca et al., 2020). Thus, PHA metabolism is one of the key metabolic strategies of *Iodobacter* sp. PCH194 to counter the multiple stresses in the Himalayan kettle lake.

Since the high-altitude Himalaya receives high amounts of UV irradiation (Singh and Singh, 2004), the inhabitant microflora must have adaptive strategies to deal with it. Consistently, *Iodobacter* sp. PCH194 possesses genes for the excinuclease system and deoxyribodipyrimidine photolyase to repair UV-induced DNA damage, as explained in the high-altitude bacterium *Acinetobacter* sp. Ver3 (Kurth et al., 2015). Additionally, PCH194 synthesizes violacein pigment as a UV protectant. The physiological experiment showed that violet-pigmented bacterium cells are more tolerant to UV (Figure 5D) than non-pigmented cells, demonstrating better survival upon adding methanol-extracted violacein pigment (Figure 5E). A literature survey supports the photoprotective role of violacein for *Janthinobacterium* spp. under UVB and UVC exposure (Abboud and Arment, 2013; Mojib et al., 2013). *Iodobacter* sp. PCH194 forms a violet microbial mat containing violacein at the surface of the medium. The phenomenon can be explained because violacein biosynthesis requires O_2_, which would be higher on the surface than in sediments. Apart from UV tolerance, violacein production and biofilm formation may be responsible for protecting the bacterium from other environmental stresses, similar to *Janthinobacterium lividum* DSM1522 (Pantanella et al., 2007). Violacein is also known as a defense against bacterivorous nanoflagellates and other planktonic community members (Matz et al., 2004; Deines et al., 2009; Batista et al., 2017) and has a strong antibacterial effect against gram-positive bacteria (Kumar et al., 2021). Violacein synthesis also served as a mechanism of interspecies interaction in *Chromobacterium violaceum* (Lozano et al., 2020). Thus, violacein production and biofilm formation constitute essential survival strategies of Himalayan *Iodobacter* spp. against abiotic and biotic stresses in its high-altitude lake habitat.

Many genomic traits of *Iodobacter* sp. PCH194 (Table 2) that were not experimentally validated might play important roles in survival under different environmental stresses. For instance, *Iodobacter* sp. PCH194 possesses genes encoding high-affinity terminal oxidases, suggesting its ability to harvest O_2_ at low concentrations for respiration (Morris and Schmidt, 2013). Genes encoding for periplasmic nitrate reductases, fumarate reductases, and transcriptional regulator (FNR) suggested that Himalayan *Iodobacter* can use nitrate or fumarate as a terminal electron acceptor under complete or near anaerobic conditions (Stewart et al., 2002). FNR is reported to act as an oxygen sensor and a molecular switch between aerobic and anaerobic respiration (Reinhart et al., 2008). Thus*, Iodobacter* has the genetic machinery for living under low oxygen concentrations and adapting to oxic-anoxic fluctuations at the sediment–water interface of the kettle lake. The various efflux pumps, porter and antiporters, and enzymes for amino acid-dependent acid tolerance might provide a survival advantage under acidic conditions (Guan and Liu, 2020). Furthermore, the toxin-antitoxin (TA) system encoded in this bacterium's chromosomal and plasmid DNA might play a crucial role in cell persistence, biofilm formation, tolerance to antibiotics, and other environmental stresses, as observed in many microbes (Page and Peti, 2016). The presence of plasmids and genes for conjugal transfer indicates the exchange of genomic information. Studies have demonstrated plasmids' adaptive, defensive, and metabolic roles in psychrophilic and psychrotolerant bacteria (Dziewit and Bartosik, 2014; Ciok et al., 2018).

## Conclusion

The high-altitude Himalaya imposes numerous stresses on its bacterial communities. Therefore, it is vital to understand bacterial adaptation to the extreme environments of the Himalayas. The bacterium *Iodobacter* sp. PCH194 possesses multiple genomic and metabolic traits, which might enable its survival under cold and freezing temperatures, nutrient fluctuations, and high UV irradiation (Figure 6). Survival at subzero temperatures was further supported by antifreeze activity of the cell extract and a differential proteome response to cold and freezing conditions. PHB synthesis under different physicochemical conditions supports adaptation to fluctuating substrate and nutrient availability. Violacein pigment protects against UV radiation. The genomic and metabolic traits found in *Iodobacter* sp. PCH194 explain its survival under challenging high-altitude conditions and provide opportunities for exploring biotechnological applications and microbial community interactions.

## Data Availability Statement

The datasets presented in this study can be found in online repositories. The names of the repository/repositories and accession number(s) can be found in the article/Supplementary Material.

## Author Contributions

DS conceived the study. VK, VT, and DS did the field trip and sampling. VK, PK, and DS designed the study and analyzed the data and wrote and finalized the manuscript. VK performed microbial, genomic, and physiological experiments. PK performed proteomic and AFP experiments. VT and SuK assisted in genomic and physiological experiments, respectively. SaK did scientific discussion and research support. All authors reviewed and approved the final manuscript.

## Funding

The study was funded by Science and Engineering Research Board (SERB), Department of Science and Technology (DST), Government of India under the scheme of National Post-Doctoral Fellowship by Grant Nos. PDF/2016/000508 and PDF/2016/003805, Scheme for Young Scientist and Technologist (SYST) Grant No. SP/YO/2019/1261. The study was generously funded to DS by the Council of Scientific and Industrial Research (CSIR), New Delhi, India with a Grant No. MLP0143 under the Niche Creating Project scheme.

## Conflict of Interest

The authors declare that the research was conducted in the absence of any commercial or financial relationships that could be construed as a potential conflict of interest.

## Publisher's Note

All claims expressed in this article are solely those of the authors and do not necessarily represent those of their affiliated organizations, or those of the publisher, the editors and the reviewers. Any product that may be evaluated in this article, or claim that may be made by its manufacturer, is not guaranteed or endorsed by the publisher.
