# Supplementary material for: Multiple Adaptive Strategies of Himalayan Iodobacter sp. PCH194 to High-Altitude Stresses
Source: Front Microbiol. 2022 Jul 6;13:881873. doi: 10.3389/fmicb.2022.881873 (PMC9298515; doi:10.3389/fmicb.2022.881873)
Supplement: Supplementary file 1 [file Table_1.DOC]

**Data Set 1: Genomic traits of *Iodobacter* sp. PCH194 for adaptation in Himalayan kettle lake**

**Genes encoding for nutritional starvation, fermentation, and PHA metabolism**

| **Description** | **Copy** | **GenBank ID** |
| --- | --- | --- |
| **PHA metabolism** |  |  |
| Class I poly(R)-hydroxyalkanoic acid synthase (*phaC*)  Poly(3-hydroxyalkanoate) synthetase (*phaC*) | 1  1 | QBC44267  QBC43446 |
| Acetyl-CoA -acyltransferase (thiolase) (*phaA*) | 2 | QBC44268, QBC42316 |
| 3-Hydroxybutyryl-CoA dehydrogenase (*phaB*) | 1 | QBC43128 |
| Beta-ketoacyl-ACP reductase/ acetoacetyl-CoA reductase (*phaB*) | 1 | QBC45234 |
| Polyhydroxyalkanoate synthesis repressor (*phaR*) | 1 | QBC45279 |
| Phasin family protein | 2 | QBC45278, QBC45322 |
| Poly(3-hydroxybutyrate) depolymerase (*phaZ*) | 1 | QBC42116 |
| Enoyl CoA hydratase (*FadB* or *phaJ*) | 3 | QBC42161, QBC43447  QBC44936 |
| **Nutritional starvation response** |  |  |
| Carbon starvation protein A | 2 | QBC44150, QBC44636 |
| Bifunctional ppGpp synthetase/guanosine-3,5-bis(diphosphate)-3-pyrophosphohydrolase | 1 | QBC44376 |
| ClpXP protease specificity-enhancing factor | 1 | QBC43777 |
| ATP-dependent Clp protease (*clpX*) | 1 | QBC42344 |
| ATP-dependent Clp endopeptidase (*clpP*) | 1 | QBC42345 |
| ATP-dependent chaperone (*ClpB*) | 1 | QBC42403 |
| ATP-dependent Clp protease (*clpA*) | 1 | QBC43216 |
| Phosphate starvation-inducible protein (*psiF*) | 1 | QBC44262 |
| **Fermentation** |  |  |
| Bifunctional acetaldehyde-CoA/ alcohol dehydrogenase | 1 | QBC45338 |
| NADH-dependent alcohol dehydrogenase | 2 | QBC43336, QBC45481 |
| S-(Hydroxymethyl)glutathione dehydrogenase/class III alcohol dehydrogenase | 1 | QBC44209 |
| Acetolactate synthase small subunit |  |  |
| Biosynthetic-type acetolactate synthase large subunit |  |  |
| D-lactate dehydrogenase | 1 | QBC45224 |
| Acetate kinase | 2 | QBC43448, QBC43516 |

**Genes for respiration under oxygen stress**

| **Description** | **Copy** | **GenBank ID** |
| --- | --- | --- |
| NADH-quinone oxidoreductase  subunit A, B, C, D, E, F, G, H, I, J, K, L, M, N  (*nuoABCDEFGHIJKLMN*) | 1 each | QBC42860, QBC42859, QBC42858, QBC42857, QBC42856, QBC42855, QBC42854, QBC42853, QBC42852, QBC42851, QBC42850, QBC42849, QBC42848, QBC42847 |
|
|
|
| Cytochrome *c*_5_ family protein | 1 | QBC42687 |
| Cytochrome *c* oxidase, *Cbb*_3_ | 1 | QBC42936 |
| Cytochrome-*c* oxidase, *Cbb*_3_-type subunit III (*CcoP*) | 1 | QBC45156 |
| Cytochrome-*c* oxidase, *Cbb*_3_-type subunit II (*CcoO*) | 1 | QBC45787 |
| Cytochrome-*c* oxidase, *Cbb*_3_-type subunit I (*CcoN*) | 1 | QBC45157 |
| CcoQ/FixQ family *Cbb*_3_-type cytochrome *c* oxidase (*CcoQ*) | 1 | QBC45786 |
| Cytochrome *c* oxidase accessory protein (*CcoG*) | 1 | QBC45155 |
| Cytochrome *c* oxidase subunit I (*ctaD*) | 1 | QBC43397 |
| Cytochrome *c* oxidase subunit II (*CoxB*) | 1 | QBC43396 |
| Cytochrome *c* oxidase subunit III | 1 | QBC43400 |
| Cytochrome *c* | 3 | QBC43524, QBC44531, QBC44601 |
| Cytochrome *b* | 3 | QBC43779, QBC42745, QBC43779 |
| Cytochrome *d* terminal oxidase subunit I (*CydA*) | 1 | QBC44810 |
| Cytochrome *d* ubiquinol oxidase subunit II (*CydB*) | 1 | QBC44811 |
| Cytochrome *bd* oxidase subunit I (*CydX*) | 1 | QBC44812 |
| Cytochrome *bd* ubiquinol oxidase subunit II | 1 | QBC44774 |
| Fumarate/nitrate reduction transcriptional regulator Fnr (*fnr*) | 1 | QBC45163 |
| Fe-S cluster assembly transcriptional regulator (*iscR*) | 1 | QBC44662 |
| IscS subfamily cysteine desulfurase (*iscS*) | 1 | QBC44661 |
| Two-component system response regulator NarL (*narL*) | 1 | QBC42122 |
| Periplasmic nitrate reductase subunit alpha (*napA*) | 1 | QBC42119 |
| Periplasmic nitrate reductase e transfer (*napB*) | 1 | QBC45585 |
| Cytochrome c-type protein (*napC*) | 1 | QBC42118 |
| Nitrate reductase (*napD*) | 1 | QBC42120 |
| Ferredoxin-type protein (*napF*) | 1 | QBC45586 |
| Nitrite reductase, copper-containing (*nirK*) | 1 | QBC44532 |
| Nitric oxide reductase large subunit | 1 | QBC44534 |
| Fumarate reductase subunit D (*frdD*) | 1 | QBC45348 |
| Fumarate reductase subunit C (*frdC*) | 1 | QBC45349 |
| Fumarate reductase iron-sulfur subunit (*frdB*) | 1 | QBC45350 |
| Fumarate reductase (quinol) flavoprotein (*frdA*) | 1 | QBC45351 |
| Arsenical-resistance protein/Ars efflux transporter (*arsB*) | 1 | QBC45711 |
| Arsenate reductase (glutaredoxin) (*arsC*) | 1 | QBC45312 |
| Organoarsenical effux MFS transporter (*arsJ*) | 1 | QBC44010 |
| Anaerobic ribonucleoside-triphosphate reductase (*nard*) | 1 | QBC45682 |

**Genes encoding for general stress, cold, UV and oxidative stress**

| **Description** | **Copy** | **GenBank ID** |
| --- | --- | --- |
| **General stress** | | |
| Universal stress protein | 1 | QBC42502 |
| General stress protein (CsbD) | 1 | QBC43198 |
| Stress responsive protein | 1 | QBC45099 |
| Stress responsive alpha-beta barrel | 1 | QBC45629 |
| Stress-responsive transcriptional regulator | 1 | QBC43637 |
| Peroxide stress protein (YaaA) | 1 | QBC43867 |
| Stress response translation initiation inhibitor (yciH) | 1 | QBC44207 |
| Stress protection protein (MarC) | 1 | QBC44499 |
| **Cold stress** | | |
| Cold-shock protein | 4 | QBC43213, QBC43214  QBC44147, QBC44547 |
| Molecular chaperone (*DnaJ*) | 2 | QBC42148, QBC43144 |
| Molecular chaperone (*DnaK*) | 1 | QBC43146 |
| Molecular chaperone (*HtpG*) | 1 | QBC42726 |
| Molecular chaperone (*SurA*) | 1 | QBC43955 |
| Molecular chaperone | 1 | QBC44670 |
| Hsp33 family molecular chaperone (*HslO*) | 1 | QBC42947 |
| Co-chaperone (*GroES*) | 1 | QBC44358 |
| Fe-S protein assembly chaperone (*HscA*) | 1 | QBC44657 |
| Fe-S protein assembly co-chaperone (*HscB*) | 1 | QBC44658 |
| Chaperonin (*GroEL*) | 1 | QBC44357 |
| ATP-dependent chaperone (*ClpB*) | 1 | QBC42403 |
| Protein-export chaperone (*SecB*) | 1 | QBC44893 |
| Fatty acid desaturase (*desA*) | 3 | QBC44097, QBC44899,  QBC45153 |
| Sterol desaturase | 1 | QBC45785 |
| **Oxidative stress** | | |
| Redox-sensitive transcriptional activator (*SoxR*) | 1 | QBC43044 |
| Oxidative damage protection protein | 1 | QBC43675 |
| Superoxide dismutase [Fe] | 1 | QBC44067 |
| Superoxide dismutase [Cu-Zn] SodC2 | 1 | QBC45525 |
| Catalase | 1 | QBC44562 |
| Thiol peroxidase | 2 | QBC42563, QBC42564 |
| Peroxiredoxin (*ahpC*) | 3 | QBC42609, QBC43928, QBC44214 |
| Organic hydroperoxide resistance protein | 1 | QBC42782 |
| Glutathione peroxidase | 2 | QBC42874, QBC44216 |
| Peroxide stress protein YaaA | 1 | QBC43867 |
| Alkyl hydroperoxide reductase subunit F | 1 | QBC44215 |
| Alkyl hydroperoxide |  | QBC44876 |
| Peroxidase | 1 | QBC44878 |
| Thioredoxin | 1 | QBC43628 |
| Thiol reductase thioredoxin | 1 | QBC44710 |
| Monothiol glutaredoxin, Grx4 family | 1 | QBC43999 |
| Glutaredoxin 3 (*grxC*) | 1 | QBC44894 |
| DNA oxidative demethylase (*AlkB*) | 1 | QBC42646 |
| **Osmotic stress** | | |
| Glycine/betaine ABC transporter ATP-binding protein | 1 | QBC44750 |
| Choline ABC transporter permease | 1 | QBC44751 |
| Glycine/betaine ABC transporter substrate-binding protein | 1 | QBC44752 |
| Glycine/betaine ABC transporter permease | 1 | QBC44753 |
| Osmotic-shock protein | 1 | QBC42974 |
| Transcriptional activator (*NhaR*) | 1 | QBC44622 |
| OsmC family protein (OsmC) | 1 | QBC44181 |
| OsmC domain/YcaO domain-containing protein | 1 | QBC44333 |
| Transcriptional regulator (*betI*) | 1 | QBC45451 |
| Betaine-aldehyde dehydrogenase (*betA*) | 1 | QBC45452 |
| Choline dehydrogenase (*betB*) | 1 | QBC45453 |
| **UV stress response** | | |
| Excinuclease ABC subunit (*UvrA*) | 1 | QBC42900 |
| Excinuclease ABC subunit B (*UVrB*) | 1 | QBC44109 |
| Excinuclease ABC subunit C (*UVrC*) | 1 | QBC45264 |
| DNA helicase II/UvrD-helicase domain-containing protein | 1 | QBC43063 |
| ATP-dependent DNA helicase Rep/UvrD-helicase domain-containing protein | 1 | QBC43169 |
| Deoxyribodipyrimidine photolyase (*phr*) | 1 | QBC43309 |

**Genes encoding for chemotaxis and motility**

| **Description** | | **Gene** | **Copy** | **GenBank ID** |
| --- | --- | --- | --- | --- |
| **Chemotaxis** | |  |  |  |
| Chemotaxis protein | | *cheA* | 3 | QBC42329, QBC45763, QBC44794 |
| Methyl-accepting chemotaxis protein | |  | 8 | QBC42325, QBC42330, QBC42698, QBC42955, QBC42966, QBC43145, QBC44497, QBC44517 |
| Chemotaxis protein | | *cheW* | 3 | QBC42326, QBC42331, BC44795, |
| Chemotaxis protein | |  | 2 | QBC45599, QBC43075 |
| Chemotaxis response regulator | |  | 2 | QBC42332, QBC44799 |
| Chemotaxis protein | | *cheR* | 2 | QBC45600, QBC44797 |
| Chemotaxis protein | | *cheY* | 1 | QBC44786 |
| Chemotaxis protein | | *cheD* | 1 | QBC44798 |
| ABC transporter substrate-binding protein | |  | 2 | QBC42693, QBC45670 |
| Dipeptide ABC transporter ATP-binding protein | | *dppD* | 1 | QBC43414 |
| Dipeptide ABC transporter ATP-binding protein | | *dppC* | 1 | QBC43415 |
| **Flagellar proteins** |  | |  |  |
| Flagellar biosynthesis protein | *fliW* | | 1 | QBC42297 |
| Flagellar biosynthesis anti-sigma factor | *flgM* | | 1 | QBC42301 |
| Flagellar motor protein | *motABCD* | | 1 each | QBC42654, QBC43203, QBC44130, QBC44990 |
| RNA polymerase sigma factor | *fliA* | | 1 | QBC44131 |
| Flagellar motor switch protein | *fliMNOPQ RSEFG* | | 1 each | QBC44141, QBC44142  QBC44143, QBC45728  QBC44144, QBC44145, QBC45008, QBC44801, QBC44802, QBC44803 |
| Flagellar protein export ATPase | *fliI* | | 1 | QBC44805 |
| Flagellar biosynthesis protein | *flhH, flhA* | | 1 each | QBC44136, QBC44137 |
|  | *flhB* | | 2 | QBC44138, QBC45012 |
| Flagellar basal body rod protein | *flgBCDEFGH* | | 1 each | QBC44700, QBC44701  QBC44702, QBC44703  QBC44704, QBC44705, QBC45757 |
| Flagellar motor switch protein | *flgI* | | 1 | QBC44706 |
| Flagellar assembly peptidoglycan hydrolase | *flgJ* | | 1 | QBC44707 |
| Flagellar hook-associated protein | *flgK* | | 1 | QBC44708 |
| Flagellar hook-associated protein 3 | *flgL* | | 1 | QBC44709 |

**Genes encoding for exopolysaccharide synthesis and export**

| **Description** | **Copy** | **GenBank ID** |
| --- | --- | --- |
| UDP-glucose 4-epimerase (polysaccharide biosynthesis protein) | 1 | QBC43123 |
| Oligosaccharide flippase family protein | 1 | QBC43124 |
| Capsular biosynthesis protein | 1 | QBC43663 |
| Beta-3-deoxy-D-manno-oct-2-ulosonic acid transferase | 1 | QBC43664 |
| Peptidyl-prolyl cis-trans isomerase (e*psD*) | 1 | QBC44076 |
| polysaccharide export protein (*epsE*) | 1 | QBC44077 |
| Chain length determinant protein (e*psF*) | 1 | QBC44078 |
| Chain length determinant protein tyrosine kinase (*epsG*) | 1 | QBC45723 |
| Exosortase B (*xrtB*) | 1 | QBC44079 |
| EpsI family protein (*epsI*) | 1 | QBC44080 |
| GDP-mannose 4,6-dehydratase | 1 | QBC44081 |
| GDP-fucose synthetase | 1 | QBC44082 |
| UDP-glucose 6-dehydrogenase | 1 | QBC44091 |
| Mannose-1-phosphate guanylyltransferase | 1 | QBC44092 |
| GDP-mannose mannosyl hydrolase | 1 | QBC45724 |

**Glycosyl transferase (GT) family protein in the genome of *Iodobacter* sp. PCH194 as revealed by dbCAN metaserver for annotating the carbohydrate active enzymes**

| **GT family** | **Activities** | **Copy** |
| --- | --- | --- |
| GT2 | Cellulose synthase (EC [2.4.1.12](http://www.enzyme-database.org/query.php?ec=2.4.1.12)); chitin synthase (EC [2.4.1.16](http://www.enzyme-database.org/query.php?ec=2.4.1.16)); N-acetylglucosaminyltransferase (EC [2.4.1.-](http://www.enzyme-database.org/query.php?ec=2.4.1.*)); N-acetylgalactosaminyltransferase (EC [2.4.1.-](http://www.enzyme-database.org/query.php?ec=2.4.1.*)) | 4 |
| GT4 | Sucrose-phosphate synthase (EC 2.4.1.14);  α-Glucosyltransferase (EC 2.4.1.52) | 4 |
| GT 51 | Murein polymerase (EC 2.4.1.129) | 3 |
| GT9 | Lipopolysaccharide N-acetylglucosaminyltransferase (EC 2.4.1.56); heptosyltransferase (EC 2.4.-.-) | 2 |
| GT5 | UDP-Glc: glycogen glucosyltransferase (EC 2.4.1.11);  ADP-Glc: starch glucosyltransferase (EC 2.4.1.21);  NDP-Glc: starch glucosyltransferase (EC 2.4.1.242);  UDP-Glc: α-1,3-glucan synthase (EC 2.4.1.183)  UDP-Glc: α-1,4-glucan synthase (EC 2.4.1.-) | 1 |
| GT19 | Lipid-A-disaccharide synthase (EC 2.4.1.182) | 1 |
| GT26 | UDP-ManNAcA: β-N-acetyl mannosaminuronyltransferase (EC 2.4.1.-);  UDP-ManNAc: β-N-acetyl-mannosaminyltransferase (EC 2.4.1.-);  UDP-Glc: β-1,4-glucosyltransferase (EC 2.4.1.-); β-1,4-Galactosyltransferase (EC 2.4.1.-) | 1 |
| GT28 | 1,2-diacylglycerol 3-β-galactosyltransferase (EC 2.4.1.46)  1,2-diacylglycerol 3-β-glucosyltransferase (EC 2.4.1.157) | 1 |
| GT30 | CMP-β-KDO: α-3-deoxy-D-manno-octulosonic-acid (KDO) transferase (EC 2.4.99.-) | 1 |
| GT35 | Glycogen or starch phosphorylase (EC 2.4.1.1) | 1 |
| GT41 | UDP-GlcNAc: peptide β-N-acetylglucosaminyltransferase (EC [2.4.1.255](http://www.enzyme-database.org/query.php?ec=2.4.1.255)); UDP-Glc: peptide N-β-glucosyltransferase (EC [2.4.1.-](http://www.enzyme-database.org/query.php?ec=2.4.1.*)) | 1 |
| GT83 | Undecaprenyl phosphate-α-L-Ara4N: 4-amino-4-deoxy-β-L-arabinosyltransferase (EC 2.4.2.43)  Dodecaprenyl phosphate-β-galacturonic acid: lipopolysaccharide core α-galacturonosyl transferase (EC 2.4.1.-) | 1 |
| GT107 | CMP-β-KDO: β-2,4-KDO transferase (EC [2.4.99.-](http://www.enzyme-database.org/query.php?ec=2.4.99.*)); CMP-β-KDO: β-2,7-KDO transferase (EC [2.4.99.-](http://www.enzyme-database.org/query.php?ec=2.4.99.*)) | 1 |

**Violacein biosynthesis and quorum sensing**

| **Description** | **Copy** | **GenBank ID** |
| --- | --- | --- |
| **Violacein biosynthesis** | | |
| Tryptophan 2-monooxygenase (*vioA*) | 1 | QBC43860 |
| Iminophenyl-pyruvate dimer synthase (vioB) | 1 | QBC43861 |
| Monooxygenase (vioC) | 1 | QBC43862 |
| Tryptophan hydrolxylase (vioD) | 1 |  |
| violacein biosynthesis enzyme (VioE) | 1 | QBC43863 |
| **Quorum sending** | | |
| S-Ribosylhomocysteine lyase (*luxS*) | 1 | QBC44964 |
| Hypothetical protein / Autoinducer 2 sensor kinase/phosphatase (*LuxQ*) | 1 | QBC43922 |

**Genes encoding for acidic pH stress response**

| **Description** | **Copy** | **GenBank ID** |
| --- | --- | --- |
| Cation-efflux pump | 1 | QBC43347 |
| Cation transporter | 1 | QBC43351 |
| Sodium:proton antiporter | 2 | QBC43499, QBC44495 |
| Na^+^/H^+^ antiporter (*nhaC*) | 1 | QBC45789 |
| Transcriptional activator (*nhaR*) | 1 | QBC44622 |
| Gluconate:proton symporter | 1 | QBC43909 |
| Trk system potassium transporter (*trkA*) | 1 | QBC43017 |
| Potassium transporter | 3 | QBC43018, QBC43783, QBC44104 |
| Potassium channel protein | 1 | QBC43866. |
| Potassium transporter Kup (*trkD*) | 1 | QBC43934 |
| K(+)-transporting ATPase subunit F (*kdpF*) | 1 | QBC45066 |
| Potassium-transporting ATPase subunit (*kdpA*) | 1 | QBC45067 |
| K(+)-transporting ATPase subunit B (*kdpB*) | 1 | QBC45777 |
| Potassium-transporting ATPase subunit C (*kdpC*) | 1 | QBC45068 |
| Two-component system; histidine kinase (*kdpD*) | 1 | QBC45778 |
| Carbamate kinase | 1 | QBC43855 |
| Ornithine carbamoyltransferase | 2 | QBC43856, QBC44721 |
| Arginine deiminase | 1 | QBC43857 |
| Arginine-ornithine antiporter (*arcD*) | 1 | QBC43858 |
| Glutamate decarboxylase | 1 | QBC44770 |
| Glutaminase | 1 | QBC45352 |
| Sodium/glutamate symporter | 1 | QBC45630 |
| Lysine transporter (*lysE*) | 2 | QBC43854, QBC44496 |
| Lysine decarboxylase | 2 | QBC44164, QBC44961 |
| Histidine decarboxylase | 1 | QBC42617 |
| F0F1 ATP synthase (Subunits A, B, C, δ, α, γ, β, ε) | 1 each | QBC43651, QBC43652, QBC43653, QBC43654, QBC43655, QBC43656, QBC43657, QBC43658 |

**Genes encoding for toxin-antitoxin (TA) system in plasmids and chromosomal DNA of *Iodobacter* sp. PCH194**

| **TA system** | **Number of genes** | **Proteins** | **GenBank ID** |
| --- | --- | --- | --- |
| Chromosomal | 7 | Zonular occludens toxin  Toxin HipA  Aerolysin family beta-barrel pore-forming toxin  Exotoxin  Toxin-antitoxin system YwqK family antitoxin  Addiction module toxin RelE | QBC42111  QBC45588  QBC42637  QBC43800  QBC44671  QBC44916  QBC44919 |
| Plasmid1 | 2 | Type II toxin-antitoxin system HipA family | QBC45968 QBC45971 |
| Plasmid2 | 0 | - | - |
| Plasmid3 | 2 | Toxin-antitoxin system, antitoxin component | QBC45974 QBC45982 |

**Plasmids of *Iodobacter* sp. PCH194 and their encoded proteins**

| **Protein name** | **Genes** | **GenBank ID** |
| --- | --- | --- |
| **Plasmid 1** | | |
| hypothetical proteins | 50 | - |
| transposase | 2 | QBC45908; QBC45954 |
| type II toxin-antitoxin system HipA family | 2 | QBC45968; QBC45971 |
| transcriptional regulator | 1 | QBC45909 |
| replication protein A | 1 | QBC45912 |
| integrase | 1 | QBC45915 |
| DUF1016 domain-containing protein | 1 | QBC45920 |
| type I restriction-modification system subunit | 1 | QBC45969 |
| MolR family transcriptional regulator | 1 | QBC45926 |
| S200/IS605 family transposes | 1 | QBC45927 |
| Molybdate metabolism regulator domain protein | 1 | QBC45928 |
| IS3 family transposase | 1 | QBC45929 |
| Partitioning protein | 1 | QBC45970 |
| Phage tail sheath family protein | 1 | QBC45937 |
| Phage tail protein | 1 | QBC45938 |
|  |  |  |
| AAA family ATPase | 1 | QBC45950 |
| IS21 family transposase | 1 | QBC45951 |
| DNA recombinase | 1 | QBC45972 |
| Pseudogenes | 4 | - |
| **Plasmid 2** | | |
| hypothetical proteins | 51 | - |
| P-type DNA transfer protein VirB5 | 2 | QBC45839; QBC45892 |
| virB8 family protein | 2 | QBC45841; QBC45894 |
| P-type conjugative transfer protein VirB9 | 2 | QBC45842; QBC45895 |
| P-type DNA transfer ATPase VirB11 | 2 | QBC45844; QBC45897 |
| type IV secretion system protein VirD4 | 2 | QBC45902; QBC45903 |
| nuclease | 1 | QBC45852 |
| transcriptional regulator | 1 | QBC45853 |
| ParA family protein | 1 | QBC45870 |
| single-stranded DNA-binding protein | 1 | QBC45872 |
| DNA cytosine methyltransferase | 1 | QBC45882 |
| lytic transglycosylase | 1 | QBC45886 |
| type I DNA topoisomerase | 1 | QBC45888 |
| type IV secretion protein A | 1 | QBC45889 |
| type IV secretion protein C | 1 | QBC45891 |
| **Plasmid 3** | | |
| hypothetical proteins | 8 | - |
| toxin-antitoxin system, antitoxin component | 2 | QBC45974; QBC45982 |
| plasmid replicase | 2 | QBC45975; QBC45983 |
| mobilization protein | 2 | QBC45977; QBC45985 |
| conjugal transfer protein TraA | 2 | QBC45978; QBC45986 |
