# Supplementary material for: Multiple Adaptive Strategies of Himalayan Iodobacter sp. PCH194 to High-Altitude Stresses
Source: Front Microbiol. 2022 Jul 6;13:881873. doi: 10.3389/fmicb.2022.881873 (PMC9298515; doi:10.3389/fmicb.2022.881873)

**SUPPLEMENTARY TABLES AND FIGURES**

**Table S1.** Physiochemical properties of Bhoot ground kettle lake in the high-altitude region of Sach Pass, Indian trans-Himalaya.

| **S. No.** | **Sample from Bhootground lake** | **pH** | **EC**  **(µS/cm)** | **Available N (kg/ha)** | **Available P (kg/ha)** | **Available K (kg/ha)** | **UV mW/cm^2^** |
| --- | --- | --- | --- | --- | --- | --- | --- |
| 1 | Soil from ground | 4.26 | 88 | 159.9 | 11.76 | 772.27 | 50.00  42.56 |
| 2 | Lake mud sample 1 | - | - | 109.8 | 11.68 | 162.13 |
| 3 | Lake mud sample 2 | - | - | 194.4 | 25.76 | 136.53 |
| 4 | Lake mud sample 3 | 4.1 | 95 | 65.86 | 19.04 | 68.267 |  |

**Table S2.** General description of Himalayan bacterium *Iodobacter* sp. PCH194.

| **Term** | **Property** |
| --- | --- |
| Classification | Domain Bacteria  Phylum Proteobacteria  Class Betaproteobacteria  Order Neisseriales  Family *Neisseriaceae*  Genus *Iodobacter* |
| Geographic location | Near Sach Pass, District Chamba, Himachal Pradesh, India |
| Sample | Sediment sample from kettle lake |
| Latitude and longitude | 33.006831°N, 76.248209°E |
| Altitude | 4160 meter above sea level |
| Colony morphology | Colonies are round with irregular margin, thin, and butyrous, with uneven surfaces, the centers show denser growth more intense pigmentation |
| Pigmentation | Violet pigmentation |
| Cell shape | Straight, round-ended rods |
| Gram stain | Gram-negative |
| Catalase | Positive |
| Nitrate reduction | Positive |
| Oxidase | Positive |
| Sporulation | Non-spore formation |
| Temperature range | -5.0 to 25 ℃ |
| pH range | 4.0 to 10.0 |
| Optimum temperature and pH | 20 ℃ and 7.0 |
| Oxygen requirement | Facultative anaerobe |
| NaCl tolerance | 0-1% |
| Sugar utilization | Dextrose, galactose, trehalose, melibiose, sucrose, L-arabinose, mannose, glycerol, mannitol, ONPG, esculin, D-arabinose, citrate, malonate |
| Sensitive for antibiotics | Penicillin, erythromycin, chloramphenicol, ciprofloxacin, streptomycin, gentamycin. azithromycin, vancomycin, rifampicin, kanamycin, tetracycline |
| Resistant for antibiotics | Miconazole, clotrimazole |

**Table S3.** Phylo-genomic analysis of *Iodobacter* sp. PCH194 with other members of the genus *Iodobacter*.

| **Bacterium** | **Genome size (Mb)** | **Genome assembly** | **NCBI reference** | **GC (%)** | **DDH (%) identities / HSP length** | **ANI (%) Two-way** |
| --- | --- | --- | --- | --- | --- | --- |
| *Iodobacter* sp. PCH194 | 4.58 | GCA_004194535.1 | NZ_CP025781.1 | 47.50 | - | - |
| *Iodobacter* sp. BJB302 | 4.81 | GCA_002735645.1 | NZ_PDZG01000001.1 | 49.60 | 26.20 | 80.63 |
| *Iodobacter* sp. H11R3 | 3.88 | [GCA_003952345.1](https://www.ncbi.nlm.nih.gov/assembly/GCA_003952345.1) | NZ_CP034433.1 | 48.00 | 25.70 | 79.99 |
| *Iodobacter* sp. HSC-16F04 | 4.78 | [GCA_011601265.1](https://www.ncbi.nlm.nih.gov/assembly/GCA_011601265.1) | NZ_JAAOLX010000001.1 | 49.40 | 25.70 | 80.45 |
| *I. fluviatilis* NCTC11159 | 5.10 | GCA_900451195.1 | NZ_UGHR01000001.1 | 48.80 | 26.40 | 80.81 |
| *I. fluviatilis* DSM 3764 | 4.96 | GCA_004346505.1 | NZ_SMBT01000001.1 | 48.70 | 25.90 | 80.81 |

**Table S4.** Proteomic response of *Iodobacter* sp. PCH194 to cold and freezing conditions showing important proteins with differential expression under cold (4 ℃) and freezing (0 ℃) conditions.

| **Accession number**  **(NCBI)** | **Entry name** | **log_2_ fold change**  (4 vs 20 ℃) | **log_2_ fold change (**0 vs 20 ℃) |
| --- | --- | --- | --- |
| **Cold shock proteins, chaperons, and proteases** | | | |
| 489840967 | Co-chaperone GrpE | -17.84 | -0.42 |
| 489774517 | Molecular chaperone GroEL | -18.94 | -18.94 |
| 489809198 | Molecular chaperone DnaJ | 0 | 16.69 |
| 488148084 | Molecular chaperone HscA | 0 | 14.92 |
| 488143175 | molecular chaperone DnaK | -3.24 | -2.30 |
| 488184620 | Immunoglobulin A1 protease family protein | 13.04 | 0 |
| 489770536 | ATP-dependent Clp protease ATP-binding protein | 0 | 17.51 |
| 754567894 | Zinc protease | 0 | 16.69 |
| 488143915 | Protease | -13.84 | -13.84 |
| 488161986 | ATP-dependent helicase HrpA | -1.63 | -17.47 |
| 489775752 | DEAD/DEAH box helicase | -19.29 | -19.29 |
| 489804667 | ATP-dependent DNA helicase RecQ | 0 | 18.26 |
| **Peptidoglycan biosynthesis and glycosyl transferases** | | | |
| 728047441 | Anhydro-N-acetylmuramic acid kinase | 0 | 16.65 |
| 489844587 | UDP-N-acetylmuramoyl-tripeptide--D-alanyl-D-alanine ligase | -15.35 | -15.35 |
| 488141282 | Phospho-N-acetylmuramoyl-pentapeptide transferase | -17.76 | -17.76 |
| 750383585 | Murein hydrolase transporter LrgA | -15.28 | -15.28 |
| 489778492 | Murein transglycosylase | -19.02 | -19.02 |
| 488147971 | Murein transglycosylase | -19.48 | -19.48 |
| 489777254 | UDP-N-acetylglucosamine 1-carboxyvinyltransferase | 0 | 14.44 |
| 490654974 | UDP-N-acetylenolpyruvoylglucosamine reductase | -21.35 | -21.35 |
| 489870211 | Glycosyl transferase | 0 | 17.89 |
| 488163065 | Glycosyltransferase family 1 | 17.41 | 0 |
| 489841934 | Glycosyl transferase | -17.71 | -17.71 |
| 489853855 | Glycosyl transferase family 2 | -19.12 | -19.12 |
| **Ribonucleases** | | | |
| 503213592 | Ribonuclease E | 0 | 19.60 |
| 489781598 | Ribonuclease Z | 0 | 16.72 |
| 488153588 | Ribonuclease E | 0 | 16.33 |
| 489867166 | Exodeoxyribonuclease V subunit beta | 0 | 15.96 |
| 488163304 | Ribonuclease II* | -13.02 | -13.02 |
| 489867432 | Ribonuclease BN* | -14.48 | -14.48 |
| **Conjugative and secretary machinery** | | | |
| 800571623 | Pilin | 10.19 | 0 |
| 645184009 | Pilus assembly protein, partial | -21.93 | -5.23 |
| 748605481 | Pilus assembly protein PilC | -16.27 | -16.27 |
| 1914831 | PilM | -16.89 | -16.89 |
| 501494968 | PilC2 | -17.14 | -17.14 |
| 488142125 | Pilus assembly protein PilW | 0 | 18.44 |
| 489807284 | Pdhesin | -3.60 | -0.04 |
| 488143028 | Adhesin MafB | -11.24 | 5.97 |
| 502850607 | Conjugal transfer protein TraE | -17.37 | -17.37 |
| 488184374 | Type-1V conjugative transfer system mating pair stabilization family protein | 0 | 15.56 |
| 489797992 | Conjugal transfer protein TrbF | -17.22 | -0.95 |
| 489839643 | Type VI secretion protein | 0 | 17.37 |
| 515031099 | Type IV secretion protein Rhs | 0 | 12.38 |
| 489839643 | Type VI secretion protein | 0 | 17.37 |
| 489797537 | Tail protein | 15.94 | 14.46 |
| 489846662 | Tail fiber protein | 0 | 12.77 |
| 489854014 | Prophage tail length tape measure protein | 0 | 18.35 |
| 488141536 | Tail protein | -17.94 | -1.63 |
| 488171643 | Tail protein | -17.84 | -16.80 |
| 488141540 | Tail protein | -18.94 | -18.44 |

**Fig. S1.** SDS PAGE showing differentially expressed polypeptides upon low temperature treatment of *Iodobacter sp*. PCH194. The differentially expressed polypeptides are numbered (1-19). These polypeptides were excised and identified by MALDI-TOF/TOF after in-gel digestion.

**
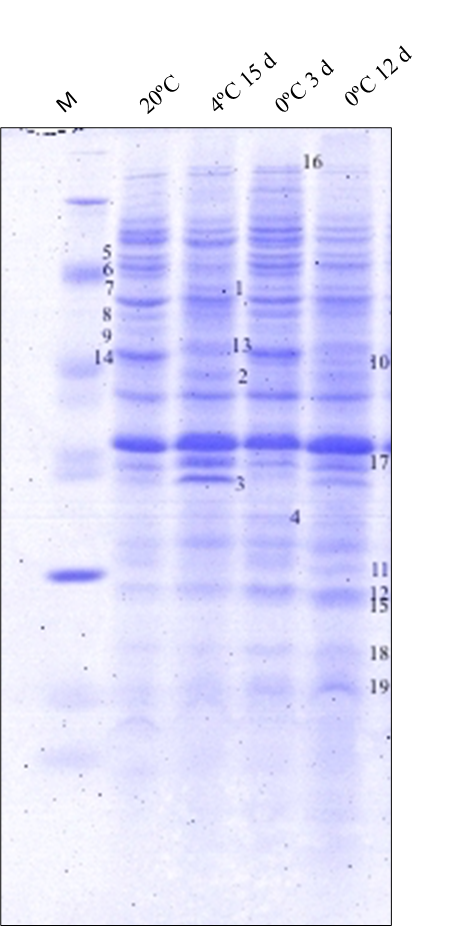
**

**Fig. S2.** Gene Ontology based categorization of proteins involved in cellular component and biological processes as identified using label-free proteomics.


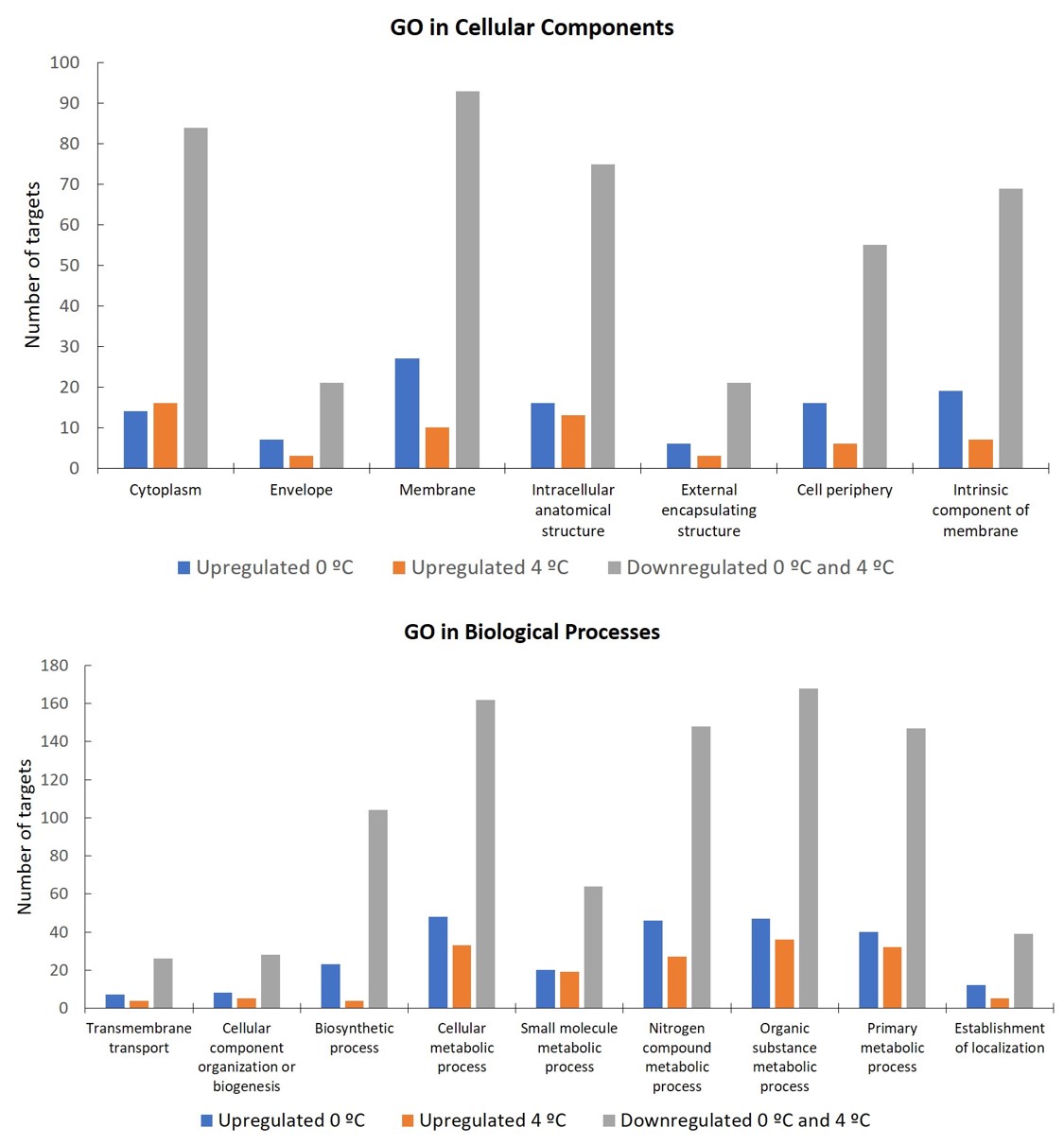

Supplement: Supplementary file 5 [file Table_5.DOC]
